# Decoupling Global and Local Structural Changes in Self-aminoacylating Ribozymes Reveals the Critical Role of Local Structural Dynamics in Ribozyme Activity

**DOI:** 10.1021/jacsau.5c00146

**Published:** 2025-05-09

**Authors:** Yu-Kai Cheng, Hsing-Hui Chu, Ning-Jun Yang, Yei-Chen Lai

**Affiliations:** Department of Chemistry, 34916 National Chung Hsing University 145 Xingda Rd., South Dist, Taichung City 402202, Taiwan

**Keywords:** self-aminoacylating ribozymes, ribozyme catalysis, divalent metal ions, local and global structural dynamics, 4-cyanotryptophan (4CNW) fluorescence

## Abstract

Self-aminoacylating ribozymes catalyze the attachment of amino acids to RNA, serving as pivotal models to investigate the catalytic roles of RNA in prebiotic evolution. In this study, we investigated how divalent metal ions (Mg^2+^ and Ca^2+^) modulate local and global structures in two such ribozymes, S-1A.1-a and S-2.1-a, using 4-cyanotryptophan (4CNW) fluorescence and native gel electrophoresis. By tracking 4CNW fluorescence changes at varying concentrations of Mg^2+^ and Ca^2+^ and temperatures, we determined how these ions influence the catalytic sites and overall conformations of the ribozymes. Our findings reveal that Mg^2+^ specifically binds to S-1A.1-a at low concentrations, stabilizing the local structure around the aminoacylation site and causing the site to become more buried, which is essential for catalytic activity. Although higher Mg^2+^ and Ca^2+^ concentrations induce global structural rearrangements, these shifts have minimal impact on the local environment of the aminoacylation site, underscoring the dominance of local structural stability in sustaining ribozyme function. In contrast, the activity of S-2.1-a effectively adapts to both Mg^2+^ and Ca^2+^, and its fluorescence results indicate a more solvent-exposed aminoacylation site. Overall, these data highlight that local structural changes in the ribozyme’s catalytic core are more critical for its function than global conformational shifts. Our study highlights the importance of local environmental changes in ion-dependent ribozyme catalysis and provides insights into the molecular mechanisms of self-aminoacylating ribozymes.

## Graphical abstract

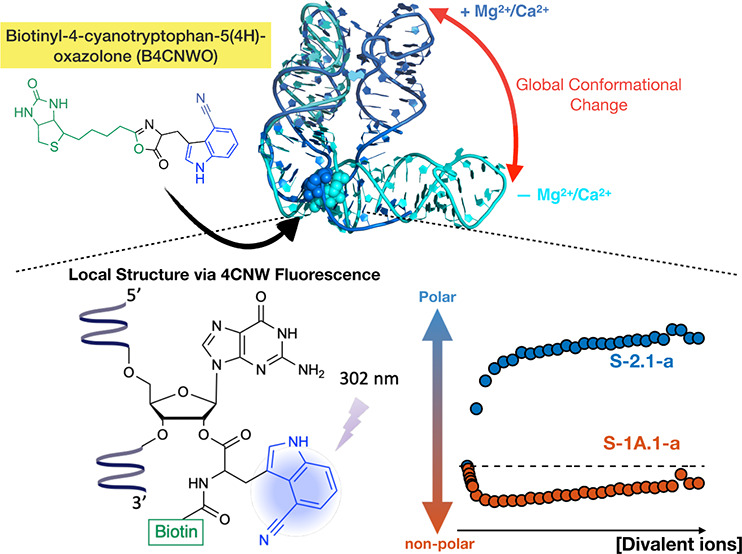

## Introduction

Ribozymes are RNA molecules capable of catalyzing specific biochemical reactions. They are essential for understanding the chemical foundations of life.^1,2^ Among these, self-aminoacylating ribozymes, which were discovered through in vitro selection, facilitate the attachment of amino acids to RNA. This process is a crucial step in exploring the origin of the protein translation. They serve as valuable models for understanding prebiotic chemistry and the catalytic roles RNA may have played during the origin of life and the evolution of the genetic code.^3–5^ Previous studies employed in vitro selection to identify two self-aminoacylating ribozymes, S-1A.1-a and S-2.1-a, that utilize aminoacyl 5­(4*H*)-oxazolone derivative, a prebiotic plausible form of chemically activated amino acids.^6,7^ S-1A.1-a and S-2.1-a have been observed to interact with aminoacyl 5­(4*H*)-oxazolone derivatives while the amino acids are selectively attached at different positions (G65 and G54, respectively). The attachment of amino acids to the specific 2′OH site is self-catalyzed by RNAs to form aminoacylated RNA. This offers insights into the mechanisms that could have driven early biochemical evolution and the transition from the RNA world to the RNA-protein world.^8,9^

Divalent metal ions, particularly Mg^2+^, are essential for the structural integrity and catalytic activity of a wide range of ribozymes. These metal ions play crucial roles across various classes of ribozymes, including self-cleaving, trans-cleaving, splicing, and ligating ribozymes.^10–12^ For example, self-cleaving ribozymes exhibit complex and diverse relationships with divalent metal ions, contributing to structural stability and catalytic function. The hammerhead ribozyme, a well-characterized self-cleaving ribozyme, relies on divalent metal ions to directly participate in catalysis. Early studies proposed mechanisms involving metal ions acting as general acids or bases, stabilizing the transition state, or coordinating the scissile phosphate.^13–16^ Recent computational work has further highlighted how ribozymes exploit electrostatic environments to recruit and position metal ions in ways conducive to catalysis, demonstrating that small self-cleaving ribozymes engineer local negative potentials in their active sites, thereby enabling site-specific metal ion binding critical for both structural stability and catalytic efficiency.^17^ The hepatitis delta virus (HDV) ribozyme depends critically on divalent metal ions for its catalytic function. The active-site cytosine residue (C75) acts as a general acid, protonating the 5′-oxygen of the leaving group, while a hydrated divalent metal ion, such as Mg^2+^, serves as a general base by stabilizing the transition state and deprotonating the 2′OH group of the ribose nucleophile.^18^ This cooperative action of C75 and the metal ion ensures efficient cleavage of the phosphodiester bond. The perturbed p*K*_a_ of C75 (∼6.1) is critical for its catalytic role, enabling it to function effectively under physiological conditions.^19,20^ Intriguingly, a recent kinetic isotope effect study revealed that the HDV ribozyme proceeds through a dissociative, metaphosphate-like transition state, highlighting the unique strategies a ribozyme can employ to facilitate catalysis.^21^ Besides the well-known hammerhead and HDV ribozymes, several recently discovered self-cleaving ribozymes—including twister, twister-sister, pistol, and hatchet ribozymes^22^—exhibit various catalytic mechanisms and interactions with divalent metal ions.^23–27^ Furthermore, DNAzymes such as the RNA-ligating 9DB1 DNAzyme, the RNA-cleaving 8–17 DNAzyme, and the 10–23 DNAzyme have demonstrated significant ion dependency in structure and function.^28–34^ Collectively, these findings underscore the multifaceted roles of divalent metal ions in ribozyme and DNAzyme activity, emphasizing the interplay of local electrostatics, metal-ion binding, and nucleic acid architecture in shaping catalytic outcomes.

In general, divalent ions serve two roles in stabilizing RNA structure. First, it screens the negative charge of the phosphate nonspecifically, enabling compact folding.^35–37^ Second, it specifically binds to nucleic acids, bringing distant bases along the RNA backbone together and forming tertiary contacts.^38,39^ The diffusive Mg^2+^ (i.e., nonspecific) usually plays a dominant role in stabilizing RNA structure.^37,40^ However, the specific binding of divalent ions has been discovered to be essential in regulating RNA function. For example, the efficiency and precision of RNA cleavage by RNase P RNA are controlled by divalent metal ions (e.g., Mg^2+^, Mn^2+^, Ca^2+^, Sr^2+^, and Ba^2+^) at various sites within the enzyme–substrate complex. The same study has demonstrated that the ratio of Mg^2+^ to Ca^2+^ adjusts the cleavage activity of RNase P RNA.^41^ The catalytic structure of RNA may also necessitate specific binding of Mg^2+^. For instance, the coordination of three Mg^2+^ ions to the specific 3′-bridging oxygens and 2′OH of the *Tetrahymena* ribozyme facilitates the formation of the catalytic site, where a phosphoryl transfer reaction occurs.^42^ Previous studies have also highlighted the essential roles of Mg^2+^ and other metal ions in aminoacyl-transferase and aminoacyl-tRNA synthetase-like ribozymes.^43–45^ These ribozymes rely on both the outer-sphere and inner-sphere coordination of metal ions to facilitate catalysis. Understanding the structural basis for this differential ion dependence is crucial for elucidating the catalytic mechanisms of ribozyme.

Due to the essential roles of divalent ions in nucleic acids, the in vitro selection of functional nucleic acids was usually performed in the presence of divalent ions to increase the probability of identifying functional species.^46,47^ However, the molecular roles of divalent metal ions in the catalytic activities of selected ribozymes are usually unclear. The two self-aminoacylating ribozymes, S-1A.1-a and S-2.1-a, were in vitro selected in the buffer containing 5 mM of Mg^2+^ and Ca^2+^. Due to the lack of structural information, the specific roles of Mg^2+^ and Ca^2+^ in modulating their catalytic activities remain unclear. While both ions share similar chemical properties, their effects on RNA folding and activity can be markedly different. Mg^2+^ is particularly well-known for its ability to induce compact and catalytically active structures in RNA molecules. In contrast, Ca^2+^, while chemically similar, often fails to induce the same catalytic conformations.^48–51^ Ca^2+^ has a larger ionic radius and lower charge density, making it more effective at screening the negative charge of the RNA phosphate backbone.^52^ Additionally, it exhibits weaker diffusive interactions with RNA compared to Mg^2+^. As a result, ribozymes may fail to adopt the precise geometries needed for catalysis in the presence of Ca^2+^.^53^ This study explores how Mg^2+^ and Ca^2+^ influence the activity and structural changes of the two ribozymes. We planned to probe local structural changes at the aminoacylation site, which is essential for understanding the core of ribozyme activity.

Strategies for probing local structures of biomolecules often involve incorporating a site-specific chemical probe at the site of interest.^54–57^ In particular, site-specific fluorescent probe modifications have been widely used in studying biomolecules due to their high sensitivity.^58^ These modifications should be introduced after synthesis to reduce the synthetic efforts required for preparing labeled biomolecules. It is preferable to target a specific site of the standard nucleotides and amino acids. Ideally, the fluorescent probe should be small and minimally perturbing to the structure and function of biomolecules. In protein structural biology studies, intrinsic amino acid fluorescence, especially tryptophan, is commonly used.^59–61^ Tryptophan is a compelling choice for this study as both ribozymes can react with the tryptophanyl 5­(4*H*)-oxazolone substrate. This allows for the probing of the aminoacylation site using the endogenous substrate.^7^ However, it is important to consider that tryptophan absorbs in the UV region, which overlaps with nucleic acid absorption and exhibits low quantum yield (<0.15) and low photostability.^62,63^ To overcome these limitations, we used 4-cyanotryptophan (4CNW), a blue fluorescent amino acid, whose absorption spectrum is red-shifted from that of the unmodified indole group on tryptophan and emits blue fluorescence.^64–66^ In addition, 4CNW demonstrates a high quantum yield, long fluorescence lifetime, and excellent photostability, rendering it a better choice than tryptophan for investigating local structural changes at the aminoacylation site. Figure 1A,B illustrates the experimental scheme for synthesizing and incorporating 4-cyanotryptophan (4CNW) into the studied ribozymes, S-1A.1-a and S-2.1-a, which were specifically labeled at their aminoacylation sites, G65 and G54, respectively.

**Figure 1:**
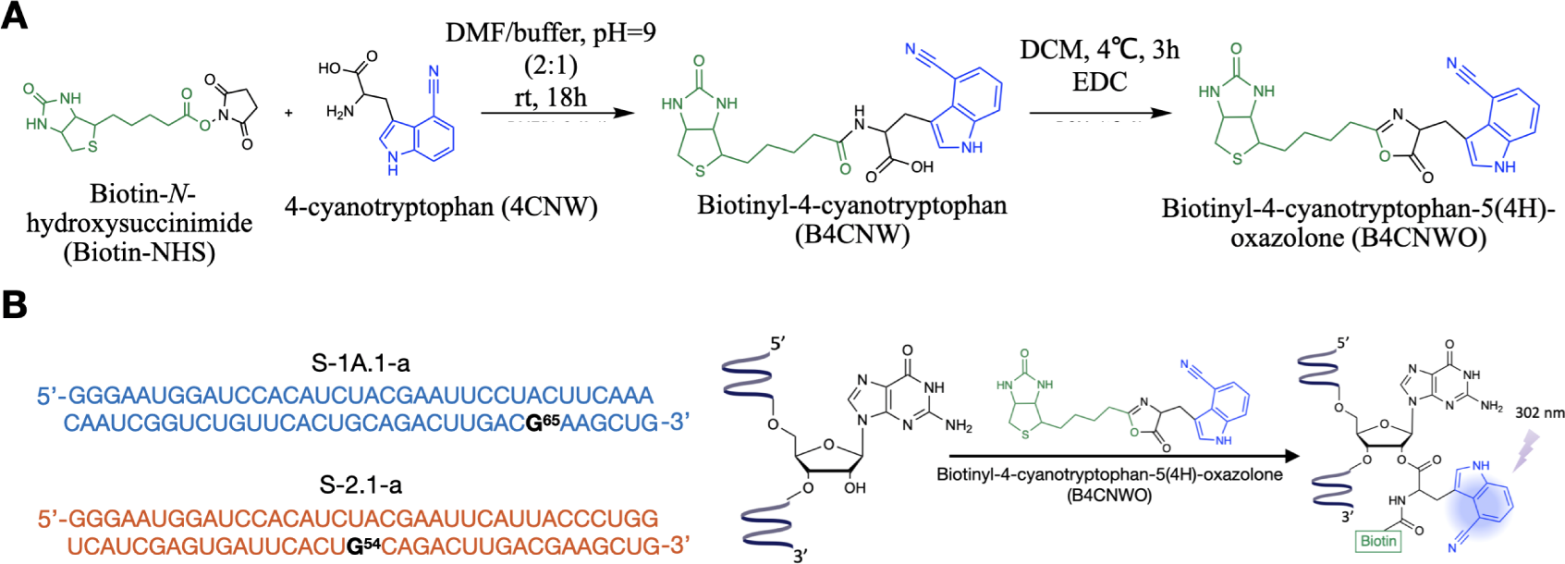
Synthesis and utilization of Biotinyl-4-cyanotryptophan-5­(4*H*)-oxazolone for probing the RNA aminoacylation site local environment. (A) Synthesis procedure of Biotinyl-4-cyanotryptophan-5­(4*H*)-oxazolone (B4CNWO). (B) Left: sequences of ribozymes S-1A.1-a and S-2.1-a, with labeling sites G65 and G54, respectively, highlighted in black. Right: B4CNWO binds to the 2′OH group of the RNA, yielding B4CNW conjugated RNA (RNA-B4CNW). The 4-cyanoindole group emits fluorescence upon excitation at 302 nm.

In this study, we used the streptavidin gel-shift assay to measure the self-aminoacylation activities of S-1A.1-a and S-2.1-a under varying Mg^2+^ and Ca^2+^ concentrations, comparing their catalytic dependencies on different ion concentrations. We then performed Mg-native PAGE to monitor potential global structural rearrangements accompanying these activity profiles. Finally, we employed 4-cyanotryptophan (4CNW) fluorescence measurements to probe how changes in divalent ion concentration and temperature affect the local environment at the aminoacylation site. By comparing the local (4CNW-based) versus global (Mg-native PAGE) conformational responses of S-1A.1-a and S-2.1-a, we seek to investigate how local structural dynamics and the RNA global folding each contribute to catalytic efficiency.

## Results

### Distinct Roles of Mg^2+^ and Ca^2+^ in Modulating S-1A.1-a and S-2.1-a Catalytic Activities

We first examined the activity of ribozymes S-1A.1-a and S-2.1-a under varying concentrations of Mg^2+^ and Ca^2+^ using a streptavidin gel-shift assay. Biotinyl-Tyr­(Me)-oxazolone (BYO), a substrate previously used in studies of these ribozymes,^6,67^ was supplied in significant molar excess (>50-fold) over the ribozymes to promote single-turnover conditions. Under these conditions, each ribozyme molecule catalyzes one self-aminoacylation event. Although the substrate is supplied in large molar excess and remains effectively high relative to the ribozyme concentration, its concentration gradually decreases over time due to continuous hydrolysis in the aqueous environment (half-life of 36.5 min).^6^ The assay measures the quantity of ribozymes that react before the substrate is lost to hydrolysis. Therefore, we define ribozyme activity as the reacted fraction (*f*) at a fixed end point of 100 min, where the reacted fraction reaches a plateau. While this end point fraction reflects both the intrinsic rate constant (*k*) and the proportion of catalytically active RNA, the extended incubation ensures that our measurement predominantly captures the fraction of ribozymes effectively achieving aminoacylation under these conditions. After the reaction, the RNA binds to streptavidin via its biotin moiety, resulting in a visible upward shift of the RNA band in the native PAGE. Figure 2A (left) shows the reacted fraction of S-1A.1-a increases as [Mg^2+^] rises, indicating enhanced activity. By contrast, Ca^2+^ alone minimally activates S-1A.1-a; however, when Ca^2+^ is present alongside Mg^2+^, it exerts a slight additional enhancement. We note that the band intensity of the SYBR gold staining in this study exhibits a linear relationship within 5–70 ng of RNA (Figure S1A). Because each lane contains 50 ng of RNA in the quantitative analysis, if the reacted fraction is below 0.1, it may fall below the detection limit. To qualitatively verify whether S-1A.1-a can react in the absence of Mg^2+^ and explore its dependency on the Ca^2+^, we increase the loading RNA amounts to 200 ng, thereby exceeding the linear range and rendering quantitative analysis less reliable. Under these conditions, we observed that S-1A.1-a can indeed react with BYO in the absence of Mg^2+^ and that its activity is enhanced at high Ca^2+^ concentrations (>50 mM) (Figure S1B). Nevertheless, the most pronounced activation occurs in the presence of Mg^2+^, highlighting its stronger effect on S-1A.1-a catalytic activity. In contrast, S-2.1-a (Figure 2B, left) exhibited increased activity with rising [Mg^2+^], yet it also showed significant activity in the absence of Mg^2+^ when Ca^2+^ was present, indicating that S-2.1-a can utilize both Mg^2+^ and Ca^2+^ as cofactors. Consistent with S-1A.1-a, we observed low activity for S-2.1-a in the absence of divalent ions in the qualitative assay (Figure S1B).

**Figure 2:**
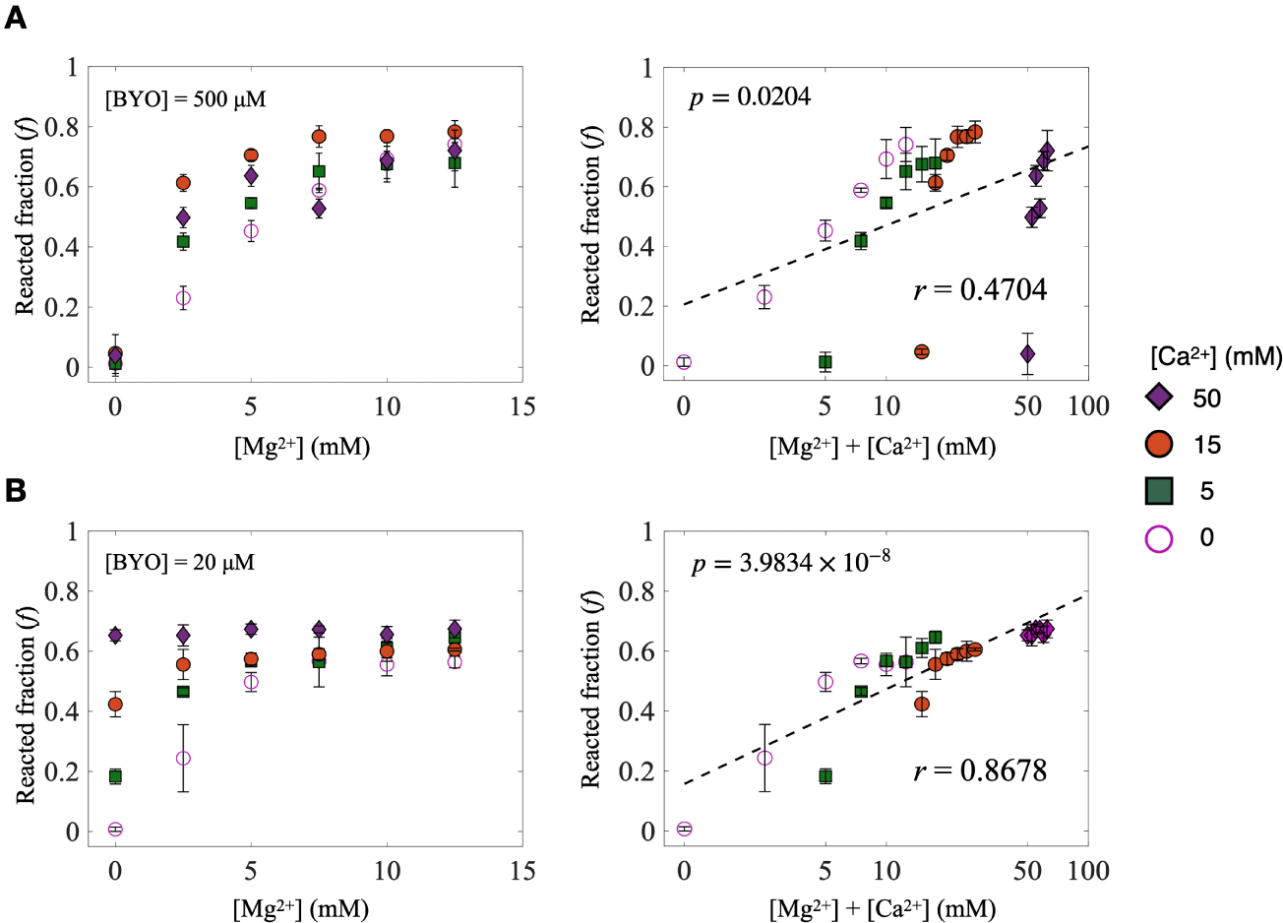
Dependence of S-1A.1-a and S-2.1-a activity on [Mg^2+^] and [Ca^2+^]. (A) Left: reacted fraction of S-1A.1-a with [BYO] = 500 μM as a function of [Mg^2+^] at different [Ca^2+^] (0, 5, 15, 50 mM). Right: the same data plotted with the sum of [Mg^2+^] and [Ca^2+^] concentrations on the *x*-axis in a logarithmic scale. (B) Left: reacted fraction of S-2.1-a with [BYO] = 20 μM as a function of [Mg^2+^] at different [Ca^2+^] (0, 5, 15, 50 mM). Right: the same data plotted with the sum of [Mg^2+^] and [Ca^2+^] concentrations on the *x*-axis in a logarithmic scale. The best linear fits (dashed line) are displayed. The calculated Pearson coefficients (*r*) and the *p*-value are shown. The error bars represent the standard deviation of the mean (*n* = 3).

To further analyze this behavior, we replotted the data with the sum of Mg^2+^ and Ca^2+^ concentrations on the *x*-axis in a logarithmic scale and the reacted fraction on the *y*-axis (Figure 2A ,B, right). The stronger linear relationship observed for S-2.1-a (Pearson coefficient, *r* = 0.8678) compared to S-1A.1-a (*r* = 0.4704) indicates that the activity of S-2.1-a is more uniformly dependent on the total concentration of Mg^2+^ and Ca^2+^. Notably, the *f* value measured at 100 min reflects the overall effectiveness of ribozyme activity under the specified conditions. It indicates the proportion of ribozymes that complete aminoacylation before the substrate is lost to hydrolysis. While the *r* value illustrates how the *f* value varies with increasing divalent ion concentrations, it does not reflect the ion dependence of intrinsic rate constants, since *f* begins to plateau at higher ion levels. We therefore interpret *r* as a comparative indicator of how efficiently divalent ions promote ribozyme self-aminoacylation across the tested concentration range. Detailed analyses for both ribozymes are shown in Figure S2. For S-1A.1-a, the *r* value for Mg^2+^ is 0.9207, indicating a strong positive linear correlation, whereas the *r* value for Ca^2+^ is 0.1413, showing a low positive correlation. For S-2.1-a, *r* values for Mg^2+^ and Ca^2+^ are 0.6373 and 0.5648, respectively, indicating moderate positive linear correlations. These results clearly suggest that the catalytic activity of S-1A.1-a specifically requires the presence of Mg^2+^. We note that in Figure 2B, the concentration of BYO was 20 μM for S-2.1-a in contrast to the 500 μM used in S-1A.1-a. This adjustment was necessary because the faster reaction rate of S-2.1-a than S-1A.1-a made it difficult to differentiate between conditions (see Figure S3). Overall, the above results indicate the different dependence of S-1A.1-a and S-2.1-a on Mg^2+^ and Ca^2+^, with S-2.1-a using both ions, but S-1A.1-a is more sensitive to Mg^2+^. The two ribozymes show different responses to divalent ions, indicating that their catalytic centers rely on divalent ions in distinct ways. In the following, we investigated this difference using the fluorescence of a 4-cyanotryptophan to study the local environment of the aminoacylation site.

### Preparing Biotinyl-4-Cyanotryptophan Conjugated RNA (RNA-B4CNW)

To probe the local environment around the ribozyme aminoacylation site of the ribozymes, we introduced 4-cyanotryptophan (4CNW) as the fluorescence probe in this study. To enable specific labeling of the aminoacylation sites of S-1A.1-a and S-2.1-a, we synthesized the substrate, biotinyl-4-cyanotryptophan-5­(4*H*)-oxazolone (B4CNWO) (Figure 1A) and verified by NMR spectroscopy and Mass spectrometry (Figures S4–S6 and Table S1). The 4CNW was synthesized following previously established methods^68,69^ and subsequently reacted with biotin-*N*-hydroxysuccinimide (Biotin-NHS) to produce biotinyl-4-cyanotryptophan (B4CNW). This intermediate was then reacted with 1-ethyl-3-carbodiimide hydrochloride (EDC) to form the B4CNWO.^6^ In Figure 1B (left), sequences of S-1A.1-a and S-2.1-a are displayed, with the labeling sites G65 and G54 (black labeled) identified as the primary positions for aminoacylation of amino acid side chains.^6,7^ The labeling reactions were carried out by ribozymes, which charged themselves with B4CNWO on the 2′OH group of the labeling sites, resulting in B4CNW conjugated RNA (i.e., RNA-B4CNW) (Figure 1B, right). The conjugation of B4CNW to the target RNAs was verified by streptavidin gel-shift assay (Figure 3A), indicating that both S-1A.1-a and S-2.1-a ribozymes can effectively use B4CNWO as a substrate for their enzymatic activities. The activity dependence of S-1A.1-a and S-2.1-a on Mg^2+^ and Ca^2+^ concentrations when using B4CNWO (Figure S7) largely mirrors the trends observed with the BYO substrate (Figure 2). Specifically, S-1A.1-a remains more responsive to increasing [Mg^2+^] than [Ca^2+^], whereas S-2.1-a efficiently utilizes both ions. Although their characteristic ion-dependence patterns remain consistent across substrates, both ribozymes display lower overall reactivity with B4CNWO than with BYO. These observations highlight inherent substrate-specific differences in these ribozymes^7^ and underscore the complex relationship between substrate identity and metal ion dependence in ribozyme catalysis.

**Figure 3:**
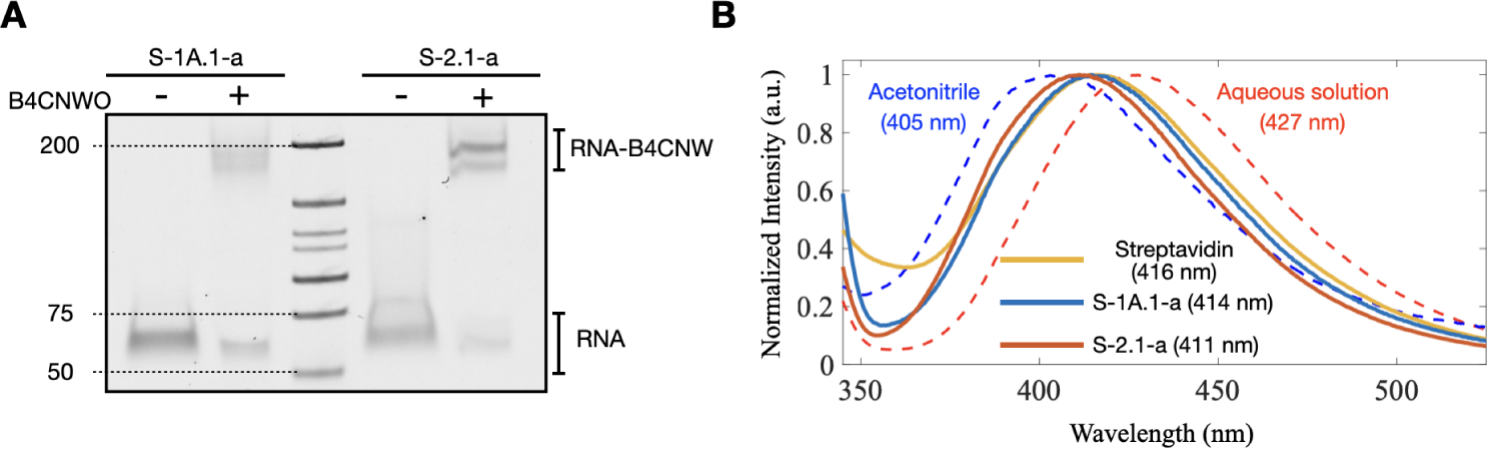
Streptavidin gel-shift assay and fluorescence spectra of B4CNW-conjugated ribozymes. (A) Streptavidin gel-shift assay results confirm the conjugation of B4CNWO to S-1A.1-a and S-2.1-a. The shift in the RNA band upon conjugation with B4CNW (RNA-B4CNW) indicates successful labeling. (B) Fluorescence spectra of 4CNW in different environments. The dashed lines represent B4CNW in acetonitrile (blue) and aqueous solutions (red). The solid lines show the fluorescence spectra of B4CNW conjugated S-1A.1-a (blue), S-2.1-a (red), and B4CNW bound to streptavidin (yellow). The wavelength of the fluorescence peak for each condition is shown. The divalent ion concentration was zero for the B4CNW-conjugated ribozyme samples. The distinct spectra demonstrate the sensitivity of 4CNW to different environments.

The fluorescence of 4CNW was observed by applying an excitation wavelength of 302 nm. We investigated the fluorescence of 4CNW under various conditions to illustrate its sensitivity to the solvent polarity and the local environment of the aminoacylation site. In acetonitrile (ACN) and aqueous solutions, the fluorescence peaks were at 405 and 427 nm, respectively (Figure 3B). These findings are consistent with previous reports on the solvent polarity dependence of the fluorescence wavelength of 4-cyanoindole,^64^ whose fluorescence red-shifts with increasing solvent polarity. Moreover, we compared the fluorescence spectra of B4CNW conjugated S-1A.1-a (blue solid line) and S-2.1-a (red solid line) and B4CNW bound to streptavidin (yellow solid line) with the peaks at 414, 411, and 416 nm, respectively. The peak wavelength of all tested conditions was lower than that of the aqueous solution condition (i.e., 427 nm), where the probe was fully exposed to the water. This indicates that 4CNW is partially embedded within the RNA structure (or the protein structure in the case of streptavidin). The noticeable shift in the peak wavelength for the local polarity of the probe indicates its ability to detect changes in solvent exposure at the RNA aminoacylation site, providing information about local structural changes within the ribozyme.

### Divalent Ions Drive Global Bent Conformations but Do Not Dictate Ribozyme Activity

To assess whether B4CNW conjugation alters the global structural equilibrium of the ribozymes, we employed the Mg-native PAGE analysis to examine the global structure changes in S-1A.1-a and S-2.1-a in the presence of Mg^2+^ and Ca^2+^. This step is crucial, as any significant alteration in global structure resulting from the conjugation would compromise the accuracy of interpreting local structural changes, potentially conflating localized effects with broader conformational shifts. The sample preparation for Mg-native PAGE does not include EDTA and hence has been used to study the kinetic and thermodynamic properties of the RNA conformation under various divalent ion buffer conditions.^70^ We analyzed the structural conformations of both ribozymes at varying Mg^2+^ concentrations (Figure 4). For the unconjugated (apo) form of S-1A.1-a, two distinct bands denoting different structural conformations were observed. Without Mg^2+^, the fast-migrating band (F1) predominated, while the slow-migrating band (S1) was faint or undetectable. According to the biased reptation theory,^71,72^ RNA migration in a gel depends on the frictional resistance caused by its shape: bent or bulged structures experience greater resistance and thus migrate more slowly than linear structures.^73,74^ As the Mg^2+^ concentration increased, the intensity of the S1 band grew significantly, reflecting a shift in equilibrium from the linear (F1) to the bent (S1) conformation. This trend was quantitatively confirmed by analyzing the intensity of the upper band, demonstrating a clear shift in the fraction from F1 to S1 with higher Mg^2+^ levels (Figure 4A, bottom). The S1 fraction, defined as the proportion of S1 within the total RNA in a lane, increases from 0.079 in 1 mM [Mg^2+^] to 0.847 in 60 mM [Mg^2+^]. This transition indicates that Mg^2+^ promotes the stabilization of the folded conformation of S-1A.1-a. Multiple bands were detected in the apo-form of S-2.1-a, indicating a broader conformational distribution compared to S-1A.1-a. Two major bands were identified in the lower and upper regions of the running lane. The fast-migrating band (F2) corresponded to the linear conformation, while the slow-migrating band (S2) represented the bent conformation. Even without Mg^2+^, a significant fraction of the S2 band was present, suggesting that S-2.1-a has an inherent tendency to adopt bent structures. As Mg^2+^ concentration increased, the intensity of the S2 band gradually increased while the F2 band diminished. The same gel intensity analysis was applied and showed that the S2 fraction sharply increased from 0.467 to 0.7 at 1 mM [Mg^2+^] and then plateaued, reaching 0.913 at 60 mM [Mg^2+^].

**Figure 4:**
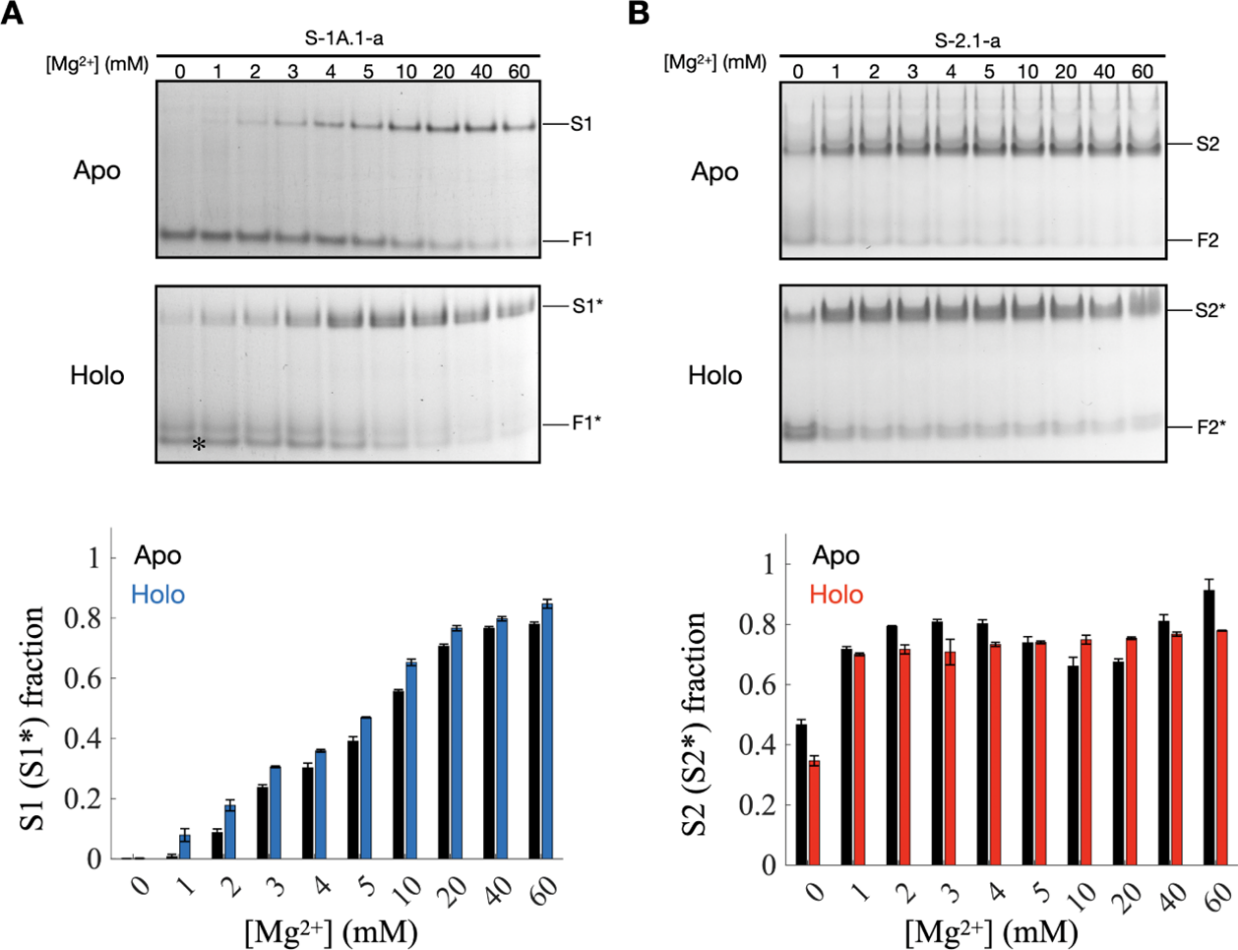
Mg^2+^-induced shifts in the structural equilibrium of S-1A.1-a and S-2.1-a ribozymes conformations. Mg-native PAGE analysis of (A) S-1A.1-a and (B) S-2.1-a at varying Mg^2+^ concentrations (0–60 mM). The top gels show the unconjugated (apo) RNA sample, and the bottom gel shows the B4CNW conjugated (holo) RNA sample. The major bands are labeled as the fast-migrating (F1, F2, F1*, and F2*) and the slow-migrating bands (S1, S2, S1*, and S2*). The asterisk in the gel denotes unreacted F1 in the holo-form sample. The bar charts below quantify the fraction of the slow-migrating band within the total RNA in a lane, representing the proportion of this structural state relative to the total population. The error bars represent the standard deviation of the mean (*n* = 3). The data indicate that both ribozymes undergo structural transitions to bent conformations in response to Mg^2+^, suggesting a shift in structural equilibrium. The results show that the conjugation of B4CNW with ribozymes does not significantly perturb the overall structural equilibrium. The error bars represent the standard deviation among triplicate measurements.

The B4CNW conjugated form (i.e., holo-form, labeled with *) of both ribozymes was prepared as described in Methods and analyzed by the Mg-native PAGE. Due to the relatively lower activity of S-1A.1-a compared to S-2.1-a, the apo-form of S-1A.1-a was present in the holo-form sample. We found that on the Mg-native PAGE, F1* and S1* can be differentiated from F1 and S1 (Figure 4A, asterisk indicated). To examine the bands observed in the native gel, the holo-form RNAs were incubated with streptavidin, resulting in a significant shift in the reacted RNAs (Figure S8). The result shows that both structures in the holo-form of the two ribozymes were conjugated with B4CNW and shifted slightly upward compared to the bands in the apo-form. For S-2.1-a, the conformational heterogeneity in the holo-form is largely reduced. Consequently, F2* and S2* exhibit sharper bands on the gel. We note that S-1A.1-a and S-2.1-a holo-forms displayed similar structural equilibrium to their respective apo-forms, with bent conformation stabilized increasingly as Mg^2+^ concentrations rose.

Interestingly, we observed almost identical results when Mg^2+^ was substituted with Ca^2+^ and several other multivalent ions (see Figures S9–S11). Besides, the stabilized bent conformation can be reversed by adding EDTA (Figure S11). While S-1A.1-a shows relatively low catalytic activity in the presence of Ca^2+^ alone, even at [Ca^2+^] = 60 mM (Figure S12A), it still exhibits structural sensitivity to Ca^2+^. These results show that although both ribozymes undergo global structural equilibrium to the bent conformation in response to increasing the concentration of Mg^2+^ and Ca^2+^, the global structural sensitivity to divalent ions does not necessarily correlate with catalytic activity. Although Mg-native PAGE reveals the global conformational dynamics of the ribozymes, it does not provide insight into local structural changes at the catalytic site, which are likely the primary determinants of activity. To further investigate this, we prepared S1A-C33G, an inactive mutant of S-1A.1-a, which lacks catalytic activity. Remarkably, this mutant displayed an identical conformation shift toward the bent structure upon adding Mg^2+^ and Ca^2+^, similar to the wild-type ribozyme (Figure S12B). This observation reinforces the hypothesis that local structural changes at the aminoacylation site, rather than global conformational shifts, dictate ribozyme activity.

### Probing the Local Structural Change at the Aminoacylation Site with 4-Cyanotryptophan

To gain deeper insights into the local structural changes at the aminoacylation site of S-1A.1-a and S-2.1-a, we first examined the changes in average fluorescence wavelength (Δ⟨λ⟩) of 4CNW in the holo-forms of S-1A.1-a and S-2.1-a with Mg^2+^ and Ca^2+^ titration at 25 °C (Figure 5A). The results show that as the Mg^2+^ concentrations increased, the fluorescence peak for S-1A.1-a shifted to a shorter wavelength (blue shift), while for S-2.1-a, it shifted to a longer wavelength (red shift), indicating an opposite change in the polarity of the environment near the aminoacylation site. Notably, the change eventually reached a plateau at different concentrations for each ribozyme. In S-1A.1-a, the average fluorescence wavelength decreased sharply at the beginning of the Mg^2+^ titration (i.e., <1 mM) and reached a plateau. On the other hand, the average fluorescence wavelength of S-2.1-a gradually increased and reached its plateau at around 5 mM, indicating that the aminoacylation site of S-1A.1-a is more sensitive to changes in [Mg^2+^] than that of S-2.1-a. The average fluorescence wavelength of S-1A.1-a remained nearly unchanged with Ca^2+^, while S-2.1-a showed similar trends to those in Mg^2+^ but with less pronounced shifts. The reversibility of the Mg^2+^-induced fluorescence wavelength shifts upon EDTA addition confirms that the observed fluorescence changes are directly governed by divalent ions (Figure S13).

**Figure 5:**
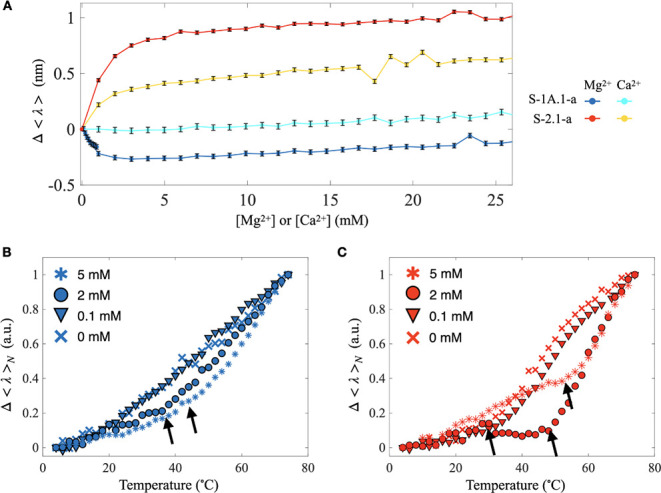
Fluorescence analysis of the local environment of the aminoacylation site in B4CNW conjugated S-1A.1-a and S-2.1-a. (A) Change in average fluorescence wavelength, Δ⟨λ⟩, of B4CNW conjugated S-1A.1-a and S-2.1-a titrated with Mg^2+^ and Ca^2+^ at 25 °C. Increasing [Mg^2+^] results in a blueshift for S-1A.1-a (blue) and a redshift for S-2.1-a (red). In contrast, Ca^2+^ titration results in minimal change for S-1A.1-a (cyan) and a relatively minor redshift for S-2.1-a (yellow). The error bars represent the standard deviation of the mean (*n* = 5). (B,C) The normalized change in average fluorescence wavelength, Δ⟨λ⟩*_N_*, for S-1A.1-a (red) and S-2.1-a (blue) at varying [Mg^2+^] (0, 0.1, 2, and 5 mM) with temperature changes. The change in slope is denoted by arrows, representing the onset of thermally induced unfolding temperature (T_*x*_), determined as described in Materials and Methods. Detailed analysis is shown in Figure S14.

We further explored the thermal stability of the aminoacylation site in the presence of different [Mg^2+^] and [Ca^2+^]. Figure 5B,C shows the normalized change in the average fluorescence wavelength (Δ⟨λ⟩*_N_*) of S-1A.1-a and S-2.1-a with varying temperatures and [Mg^2+^], respectively. In general, we observed a red shift in the fluorescence peaks as the temperature increased, indicating the exposure of the aminoacylation site. The onset of thermally induced unfolding temperature (T_*x*_) was identified when the fluorescence wavelength changed rapidly, indicating the start of the unfolding process (see Methods). For S-1A.1-a, T_*x*_ could be determined for 2 mM and 5 mM Mg^2+^ (see Figure S14) but not at lower concentrations due to the lack of an evident slope change. The T_*x*_ of S-1A.1-a increased from 39.9 °C at 2 mM to 46.7 °C at 5 mM. For S-2.1-a, T_*x*_ can be determined for all measured conditions, with T_*x*_ shifting to higher temperatures as Mg^2+^ concentration increased (27.9 °C at 0 mM, 33.1 °C at 0.1 mM, 49.0 °C at 2 mM, and 57.6 °C at 5 mM). The increase of [Mg^2+^] causes the T_*x*_ to shift to higher values, indicating the structural stabilizing effect of Mg^2+^. We found that the thermal stability of S-2.1-a improves significantly as [Mg^2+^] increases from 0.1 mM to 2 mM. In contrast, S-1A.1-a shows little sensitivity to changes in [Mg^2+^] within this concentration range. Notably, the similar T_*x*_ values obtained for S-1A.1-a and S-2.1-a in 2 mM Ca^2+^ compared to those in 2 mM Mg^2+^ (Figure S14) indicate that Ca^2+^ can also stabilize these structures.

To further investigate how Mg^2+^ alters the local environment of these ribozymes, we monitored molecular environment changes at two temperatures (15 and 25 °C). Figure 6A,B presents the relative peak fluorescence intensity and average fluorescence wavelength changes for S-1A.1-a and S-2.1-a, respectively. The blue shift observed in S-1A.1-a indicates that 4CNW becomes more buried within the RNA, which leads to dynamic fluorescence quenching through interactions (collisions) with nearby nucleobases.^60,75–77^ Indeed, S-1A.1-a’s fluorescence intensity decreases with rising [Mg^2+^], but the quenching effect is reduced at 15 °C (i.e., the signal decays more slowly), consistent with a dynamic quenching mechanism. For S-2.1-a, peak intensity also diminishes with increasing [Mg^2+^], yet we observe no correlation between quenching and temperature. This suggests that quenching in S-2.1-a mainly arises from other temperature-insensitive quenching mechanisms. Notably, guanine residues are known to quench indole and 4CNW fluorescence through photoinduced electron transfer (PET);^78,79^ thus, placing the fluorophore at G65 in S-1A.1-a or G54 in S-2.1-a predisposes it to guanine-mediated quenching whenever the fluorophore is brought into proximity with other nucleobases. Although the red shift in fluorescence wavelength for S-2.1-a indicates increased solvent exposure, the overall intensity may still decrease if guanine-based quenching becomes more efficient under these rearranged conformations, ultimately outweighing any fluorescence enhancement that might result from greater solvent accessibility. To determine whether these effects apply broadly to other divalent ions, we conducted the same assay using Ca^2+^. Similar intensity decay was observed for both ribozymes upon Ca^2+^ addition (Figure S15A,B), indicating that this quenching arises from a rather general divalent ion-induced structural reorganization rather than a Mg^2+^-specific process. We also confirmed that free B4CNW exhibits negligible fluorescence changes when exposed to Mg^2+^ (Figure S15C), demonstrating that Mg^2+^-induced structural changes in the ribozyme, rather than direct cation coordination to the fluorophore, are primarily responsible for quenching.^80^ Finally, the lack of a pronounced temperature dependence in the wavelength shift (Figure 6B, right) supports the idea that the environment near the aminoacylation site remains relatively stable over the investigated temperature range.

**Figure 6:**
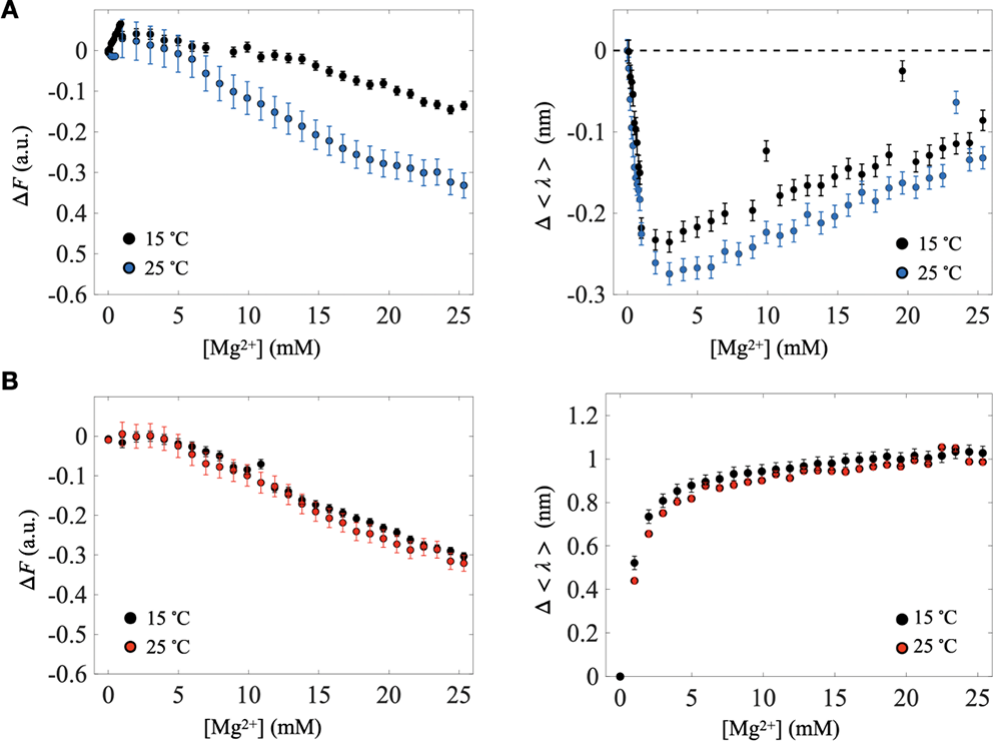
Temperature-dependent fluorescence analysis with Mg^2+^ titration. The relative peak fluorescence intensity (Δ*F*, left) and average fluorescence wavelength change (Δ⟨λ⟩, right) as a function of [Mg^2+^] at 15 and 25 °C for (A) S-1A.1-a and (B) S-2.1-a. For S-1A.1-a, the decrease in peak intensity indicates Mg^2+^-induced quenching, which is reduced at 15 °C, suggesting a dynamic quenching mechanism. The blue shift in Δ⟨λ⟩ implies the burial of the 4CNW fluorescence group within the RNA tertiary structure of S-1A.1-a supporting the dynamic quenching mechanism. For S-2.1-a, the reduction in relative peak fluorescence intensity with increasing [Mg^2+^] also indicates Mg^2+^-induced quenching but with no observable temperature dependence, suggesting a nondynamic quenching mechanism. The red shift in Δ⟨λ⟩ implies the exposure of the 4CNW fluorescence group for S-2.1-a. The trends of Δ⟨λ⟩ at two different temperatures are similar, indicating a consistent environmental change of the aminoacylation site to Mg^2+^ within this temperature range. The error bars represent the standard deviation of the mean (*n* = 5).

### Distinct Catalytic Mechanisms and Ion Sensitivities of S-1A.1-a and S-2.1-a

Fluorescence data demonstrated that the aminoacylation site of S-1A.1-a is highly responsive to low Mg^2+^ concentrations (<0.1 mM), more so than S-2.1-a. However, the thermal stability of the active structure of S-1A.1-a may be compromised at room temperature (25–27 °C) at low Mg^2+^ concentrations. We hypothesized that both ribozymes may maintain a small fraction of their active structures, even in the absence of divalent ions, and the introduction of divalent ions could shift the conformational equilibrium from an inactive to an active structure. To validate our hypothesis, we performed streptavidin gel-shift assays (Figure 7A) by increasing RNA loading to 200 ng (i.e., the qualitative assay as described) and reducing the reaction temperature to 4 °C, revealing faint but detectable aminoacylation activity for both ribozymes without Mg^2+^ and even in the presence of 1 mM EDTA. The inclusion of EDTA served to chelate any residual divalent ions in the test tube, ensuring that the observed activity was genuinely metal-ion independent. The presence of aminoacylation activity in the EDTA-treated samples suggests that the ribozymes can maintain a catalytically active conformation without divalent ions. These results suggest that the local structure folding supports catalytic activity, even when global ion-dependent folding is hampered. Furthermore, at 0.1 mM Mg^2+^, S-1A.1-a displayed clear aminoacylation activity, while S-2.1-a showed only marginal activity, underscoring the high sensitivity of S-1A.1-a to Mg^2+^. This experiment highlights the critical role of local structural changes in governing the activity of S-1A.1-a, which is more sensitive to low Mg^2+^ concentrations than S-2.1-a.

**Figure 7:**
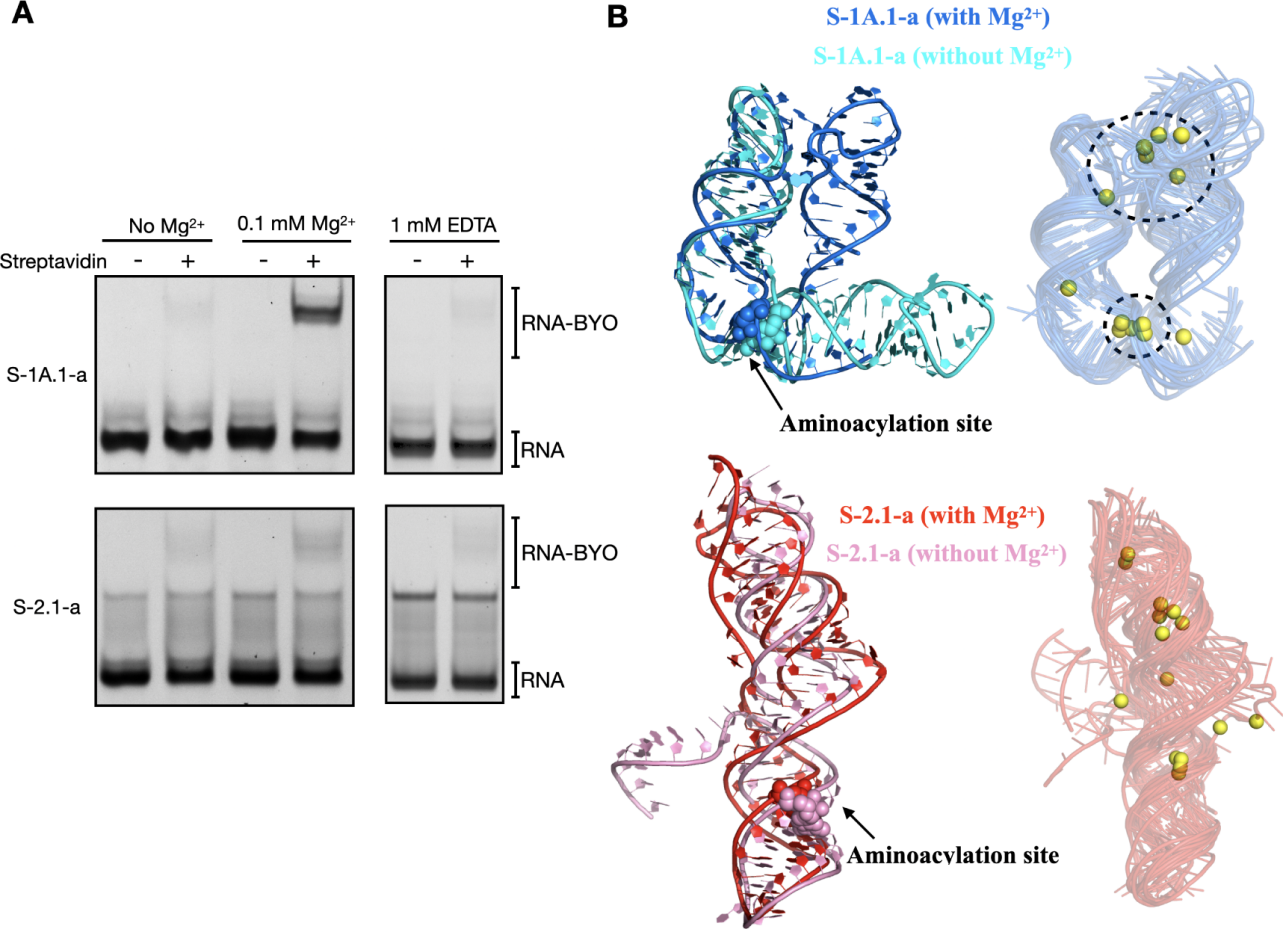
The activity of S-1A.1-a and S-2.1-a at low [Mg^2+^] and their structure predictions. (A) The streptavidin gel-shift assay shows the reacted fractions of S-1A.1-a (top) and S-2.1-a (bottom) with [BYO] = 500 μM at 4 °C in the absence of Mg^2+^, with 0.1 mM Mg^2+^, and with 1 mM EDTA. (B) AlphaFold 3 predicted structures of S-1A.1-a (top) and S-2.1-a (bottom) with (blue and red) and without (cyan and pink) Mg^2+^ are shown. The aminoacylation sites are highlighted in spheres. The predicted structures demonstrate the conformational changes induced by Mg^2+^ binding. The corresponding two-dimensional drawings created with RiboDraw^87^ are displayed in Figure S16. Ten predicted structures of each ribozyme with Mg^2+^ (yellow) from two different seeds (one with a single Mg^2+^ and the other with two Mg^2+^) were aligned. The clustered Mg^2+^ binding sites in S-1A.1-a are highlighted.

To gain insight into the possible tertiary arrangements of S-1A.1-a and S-2.1-a, we used AlphaFold 3 ^81^ to predict the tertiary folds of both ribozymes with and without Mg^2+^ ions (see Methods). We note that accurate ribozyme structures remain particularly challenging for computational prediction,^82,83^ partly because local conformations and catalytically relevant pockets can be difficult to capture.^84^ Nonetheless, AlphaFold 3 has shown promising results for short RNA sequences,^85,86^ and we leverage its global predictions here as a complementary tool rather than a definitive result.

In the predicted models, S-1A.1-a (Figure 7B) without Mg^2+^ (cyan) features an L-shaped stacking of two stem-loops, whereas the Mg^2+^-bound structures (blue) suggest a “kissing” stem-loop arrangement. We note that these outputs should not be interpreted as proof that all conformations are populated in solution; rather, they represent plausible structural scenarios illustrating how Mg^2+^ might influence tertiary contacts. Notably, AlphaFold 3 consistently places Mg^2+^ ions (yellow spheres) near the aminoacylation site G65, which resonates with our experimental observation that Mg^2+^ strongly modulates the local structure. Meanwhile, our Mg^2+^-native PAGE data (Figure 4) reveal a bent conformation at elevated Mg^2+^ concentrations, broadly compatible with AlphaFold’s folded states. In the case of S-2.1-a, the models consistently predict a coaxially stacked arrangement of stem-loops, whether Mg^2+^ is present or not (pink). In several predicting models, the pseudoknot structure is observed with and without Mg^2+^ (red); however, it appears more frequently in the presence of Mg^2+^ according to several predictions. These observations imply that while S-2.1-a’s overall stem-loop framework may remain largely intact, Mg^2+^ could favor the formation or stabilization of more complex features (i.e., pseudoknot structures). Overall, AlphaFold 3 offers a starting point for exploring how divalent ions could shape the global architectures of these two ribozymes. It suggests plausible ways in which ion binding may tip the equilibrium between more compact and more extended states, helping us form testable hypotheses about ribozyme folding under varying ionic conditions.

## Discussion

In this study, we investigated the roles of Mg^2+^ and Ca^2+^ in modulating the catalytic activities of two self-aminoacylating ribozymes, S-1A.1-a and S-2.1-a. We demonstrated that S-1A.1-a activity primarily depends on the concentration of Mg^2+^, while S-2.1-a can utilize both Mg^2+^ and Ca^2+^ as cofactors, with a stronger positive correlation with the total concentration of these divalent ions (Figure 2). This suggests that S-2.1-a is less specific to Mg^2+^. This differential ion dependence is consistent with previous studies that highlight the unique roles of Mg^2+^ in ribozyme activity and folding.^88–91^ Specific ion binding to RNA, which requires energetically costly partial dehydration and strongly depends on the ion radius, is presumed to be essential in regulating the ion binding to RNA.^35^

Our results indicate that Mg^2+^ plays a critical role in stabilizing the catalytically active conformation of S-1A.1-a, consistent with prior studies of acyl-transferase ribozymes.^92,93^ However, while previous works have shown that outer-sphere and inner-sphere coordination can facilitate catalysis, our study highlights that Ca^2+^ cannot induce the same catalytic activity level in S-1A.1-a. This suggests that the specific coordination geometry and charge density of Mg^2+^ are critical for forming the active site. The experimental results indicate a strong preference for Mg^2+^ over Ca^2+^ in S-1A.1-a, suggesting specific binding of Mg^2+^ to stabilize the catalytically active structure of S-1A.1-a. The preference for Mg^2+^ over Ca^2+^ can be attributed to its smaller ionic radius and higher charge density, which allow for a tighter interaction with both the RNA backbone and the active site residues. A notable blue shift in the 4CNW fluorescence of S-1A.1-a induced by Mg^2+^ suggests that its aminoacylation site becomes buried upon interacting with Mg^2+^. Outer-sphere interactions between divalent ions (both Mg^2+^ and Ca^2+^) and S-1A.1-a could induce additional global structural changes (i.e., S1* population increase shown in the Mg-native PAGE) while maintaining the environment of the catalytic core minimally perturbed. Our results suggest that local and global structural changes can be decoupled. For S-1A.1-a, while the global conformation can be shifted by both Mg^2+^ and Ca^2+^, the local environment at the aminoacylation site only becomes more compact in the presence of Mg^2+^, as indicated by the blue shift in the 4CNW fluorescence. In contrast, for S-2.1-a, Mg^2+^ and Ca^2+^ seemed equally beneficial to its activity, suggesting that the catalytic structure of S-2.1-a is derived mainly from diffusive divalent ions. The higher structural heterogeneity of S-2.1-a, as shown in the Mg-native PAGE (Figure 4B), also suggests that S-2.1-a can use diverse divalent ions and has more adaptable binding sites than the precise coordination required by S-1A.1-a. The flexible structure of S-2.1-a may enable it to accommodate different divalent ions, thereby maintaining catalytic activity under varying conditions.

In this study, we concentrated on the roles of Mg^2+^ and Ca^2+^ ions in ribozyme activity, despite the fact that certain ribozymes demonstrate increased catalytic activity with alternative ions such as Mn^2+^,^94^ Zn^2+^,^95^ or lanthanides^96^ owing to their distinctive coordination properties. We emphasized Mg^2+^ and Ca^2+^ due to their physiological relevance and their utilization during the in vitro selection of S-1A.1-a and S-2.1-a. Our findings highlight the significant impact of Mg^2+^ and Ca^2+^ on ribozyme activity and structure. Nevertheless, future investigations could examine the effects of other divalent ions on the catalytic efficiency and structural dynamics of these ribozymes. Such research should provide valuable insights into the adaptability and evolutionary significance of ribozyme-ion interactions.

Our results highlight the varying structural dynamics of the two ribozymes, where S-1A.1-a relies on local structural integrity for catalytic efficiency, while S-2.1-a exhibits a more flexible and exposed conformation overall. Interestingly, previous studies have shown that S-1A.1-a preferred aminoacyl oxazolone substrates with aromatic amino acid side chains. On the contrary, S-2.1-a uses a variety of aminoacyl oxazolone substrates, including those with aromatic side chains, small hydrophobic alkyl side chains (e.g., leucyl-, isoleucyl-, and valyl-), and sulfur-containing side chains (e.g., methionyl-).^6,7^ The higher promiscuity of S-2.1-a could stem from its higher structural heterogeneity, presenting the flexibility to accommodate a broader range of substrates,^10,97,98^ The flexible structure of S-2.1-a could enable it to maintain catalytic activity under varying divalent ion conditions and substrates. In contrast, S-1A.1-a exhibits a more specific dependence on Mg^2+^, possibly indicating its specialized role at the aminoacylation site of S-1A.1-a.

We showed that the unfolding temperatures (T_*x*_) of S-2.1-a and S-1A.1-a generally increase in the presence of Mg^2+^ and Ca^2+^, indicating that these divalent ions can thermodynamically stabilize the folding structures of ribozymes (see Figures 5B,C and S14). Combined with the results of Mg-native PAGE, this suggests that for S-2.1-a, stabilizing its bent structures (i.e., S2 band) by Mg^2+^ and Ca^2+^ could be essential for its function, as its catalytic activity requires about 2 mM of divalent ions (see Figures 2B and S3). This is consistent with studies on ribozymes where divalent ions binding is essential for structural integrity and catalytic activity.^99–101^ For S-1A.1-a, a similar structure stabilization effect for the divalent ions is observed, though its T_*x*_ change with respect to the [Mg^2+^] is relatively small compared to S-2.1-a. Notably, the fraction of the bent conformation of S-1A.1-a (i.e., S1 and S1*) changes significantly, increasing from less than 0.01 to approximately 0.5, with an increase in [Mg^2+^] from 0 mM to 5 mM. This observation implies that while the overall structure of S-1A.1-a is affected by Ca^2+^ and Mg^2+^, the local structure of the aminoacylation site does not seem to experience significant differences. This supports the model that S-1A.1-a can function efficiently with minimal Mg^2+^ binding, relying on specific local structural adjustments rather than extensive global rearrangements. This behavior is similar to ribozymes that utilize specific local interactions with Mg^2+^ for their catalytic functions. For example, the CPEB3 self-cleaving ribozyme adopts its globular folded structure in monovalent ions alone, but up to eight Mg^2+^ binding sites are crucial for forming the active structure.^102^

Our experimental characterization relies on using 4CNW as a fluorescent probe, providing insights into the local environment of the aminoacylation site of the ribozymes. By monitoring its fluorescence changes in response to varying concentrations of Mg^2+^ and Ca^2+^, we could distinguish between global and local structural changes. The blue shift observed in S-1A.1-a during Mg^2+^ titration suggests that the fluorophore becomes more buried within the RNA, leading to dynamic quenching by nucleobases.^60,75–77^ We show that the aminoacylation site of S-1A.1-a is structurally buried, whereas it is relatively exposed for S-2.1-a. This information clarifies the catalytic mechanism of these two ribozymes. The specific binding of Mg^2+^ to S-1A.1-a leads to the catalytic core formation, optimizing its interaction with specific substrates. In contrast, S-2.1-a, with its broader substrate specificity and less specific ion dependence, likely maintains a more flexible and adaptable active site, allowing it to function under various ionic conditions and substrates. Our data suggest that local structural environments play a significant role in the catalytic efficiency of ribozymes and that understanding these local changes can lead to a better grasp of ribozyme functionality.

## Conclusion

This study investigated the impact of Mg^2+^ and Ca^2+^ on the catalytic activities of self-aminoacylating ribozymes, S-1A.1-a and S-2.1-a, using biochemical assays and fluorescence spectroscopy with 4CNW. Our results show that S-1A.1-a favors Mg^2+^ over Ca^2+^ and remains active at low Mg^2+^ concentrations, highlighting the unique ability of Mg^2+^ to stabilize its active conformation through tight binding and effective charge neutralization. The 4CNW fluorescence data indicate that Mg^2+^ induces local structural changes, leading to a buried aminoacylation site for S-1A.1-a. In contrast, S-2.1-a efficiently utilizes both Mg^2+^ and Ca^2+^, exhibiting relatively broader ion adaptability. The 4CNW fluorescence results suggest a relatively exposed aminoacylation site of S-2.1-a, reflecting its structural flexibility observed in the Mg-native PAGE analysis. This study emphasizes the significance of local environmental changes in ribozyme function and provides insights into ion-dependent catalysis. Understanding these mechanisms is crucial for elucidating the evolutionary significance of divalent ions in RNA-based life forms and underscores the need for further experimental exploration of localized ribozyme-ion interactions.

## Materials and Methods

### Materials and General Methods

All chemicals were obtained from commercial resources and used without further purification. The NMR spectra were recorded on Agilent DD2 (600 MHz) and Varian-400 MR (400 MHz) NMR spectrometers with CDCl_3_, d6-DMSO, and D_2_O as the solvent. Mass spectra were collected using an UltrafleXtreme MALDI-TOF/TOF mass spectrometer from Bruker Daltonics, equipped with an Nd: YAG laser (λ = 355 nm) and controlled by FlexControl data collection software. The mass spectra were acquired by summing 1000 single-shot mass spectra. DNAs were chemically synthesized and were page purified by Pro-Tech chemicals, Taiwan. Biotinyl-Tyr­(Me)-oxazolone (BYO) was prepared as previously described.^6^ Before any assays, RNA samples were heat-refolded in a buffer without Mg^2+^ or Ca^2+^ at 65 °C for 5 min and then cooled to room temperature.

### Synthesis of Biotinyl-4-Cyanotryptophan-5­(4*H*)-Oxazolone

To a solution of 4CN-Trp (0.1146 g, 0.5 mmol) in 2.0 mL of 100 mM sodium phosphate, pH 8.0, in a 25 mL round-bottom flask was added biotinyl-*N*-hydroxysuccinimide (0.1717 g, 0.5 mmol). The pH of the solution was adjusted to 9 with 1 M NaOH. After stirring at room temperature overnight, the precipitate was filtered off, and the filtrate was evaporated under reduced pressure. The product was precipitated by adjusting the remaining solution to pH 2–3 with 1 M HCl to yield biotinyl-4-cyanotryptophan (B4CNW, yellow solid). The solid was dried with phosphorus pentoxide in a vacuum. Subsequently, to a solution of EDC (0.1342 g, 0.7 mmol) in 2 mL of dichloromethane in a 25 mL round-bottom flask was added B4CNW (0.0455 g, 0.1 mmol) in 5 mL of dichloromethane. The mixture was stirred and sonicated at 4 °C for 3–4 h until all starting material was dissolved. The mixture was then washed once each with water, saturated sodium bicarbonate, and saturated brine. The organic layer was dried over anhydrous magnesium sulfate and concentrated under reduced pressure to yield biotinyl-4-cyanotryptophan-5­(4*H*)-oxazolone (B4CNWO).

### In Vitro RNA Preparation

Chemical synthesis was used to obtain DNA molecules having the sequence 5′-GATAATACGACTCACTATAGGGAATGGATCCACATCTACGAATTC-N21-TTCACTGCAGACTTGACGAAGCTG-3′; the nucleotides upstream of the transcription start site for T7 RNA transcriptase are underlined, and N21 denotes 21 consecutive nucleotides which are CTACTTCAAACAATCGGTCTG for S-1A.1-a and ATTACCCTGGTCATCGAGTGA for S-2.1-a. DNAs were PCR amplified using Pfu DNA polymerase (Bioman Scientific Co., Taipei, Taiwan). RNAs were transcribed using T7 RNA Polymerase ver.2.0 (Takara). In vitro T7 promoter-based transcription was performed overnight at 37 °C with 200 U of T7 RNA polymerase in the presence of 10 mM NTPs, 1 μg of dsDNA template, 20 U of RNase inhibitor (RNAok, SMOBIO, Taiwan), and 1X T7 RNA Polymerase buffer (Takara) included in the reagent kit. The transcribed RNA was DNase I digested at 37 °C for 15 min and then purified from the denaturing polyacrylamide gel electrophoresis. RNA was excised from the gel and eluted in storage buffer (10 mM Tris and 1 mM EDTA, pH 8.0), followed by ethanol precipitation. The RNA pellets were dissolved in 100 μL of storage buffer. The RNA concentration of each sample was determined by the absorbance at 260 nm using Biophotometer (Eppendorf).

### Determining the Fraction of Reacted Ribozymes by Streptavidin Gel-Shift Assay

Streptavidin gel-shift assays for observation of reactivity were performed with 0.4 μM RNA and 500 μM BYO or B4CNWO per sample unless otherwise noted. RNAs were incubated with substrates for 100 min at room temperature with varying concentrations of Mg^2+^ and Ca^2+^ in a HEPES buffer (100 mM HEPES, 100 mM NaCl, 100 mM KCl, pH 8.00). The reactions were stopped by removing unreacted substrate using Bio-Spin *p*-30 desalting columns (Bio-Rad) and exchanging the buffer to desalting buffer (10 mM Tris, 100 mM NaCl, pH 7.00). Subsequently, the samples (50 ng of RNAs for quantitative assay and 200 ng of RNAs for qualitative assay) were treated with 4 μM streptavidin (NEB) for 15 min and then, in a 7:3 volume ratio, mixed with a TE-loading dye buffer (10 mM Tris-HCl, 60 mM EDTA, 40% (w/v) glycerol, 0.03% (w/v) of bromophenol blue and xylene cyanol) subjected to analysis via 8% polyacrylamide (29:1 acrylamide/bis-acrylamide) gel made in TBE buffer (44.5 mM Tris, 44.5 mM Boric acid and 1.4 mM EDTA pH 8.30). The native PAGEs were stained with SYBR Gold (Invitrogen) in Tris buffer (10 mM Tris, pH 8.0) for 10 min. After staining, images were captured using the CCD camera on the UV transilluminator (SmartView 111, Major Science) and then analyzed using ImageJ software (NIH).

### Pearson Coefficient Analysis

Pearson correlation coefficients (*r*) were calculated to quantify the correlation between ribozyme activity and the divalent ion concentration. The reacted fraction was plotted on the *y*-axis, while the logarithm of the Mg^2+^, Ca^2+^ concentration, or their combined concentration, was plotted on the *x*-axis. Linear regression analysis was performed to calculate the *r*, which indicates the strength and direction of the linear relationship between ribozyme activity and divalent ion concentration. The *r* can range from −1 to 1, where a value close to 1 suggests a strong positive linear correlation, a value close to −1 indicates a strong negative linear correlation, and a value around 0 indicates no linear correlation. *p*-values were calculated to evaluate the statistical significance of these correlations.

### Mg-Native Polyacrylamide Gel (Mg-Native PAGE) Preparation and Electrophoresis

RNAs (7 pmol) in a Tris-NaCl buffer (10 mM Tris and 100 mM NaCl, pH 8.00), with specific Mg^2+^ and Ca^2+^ concentrations, were incubated with a loading dye buffer (10 mM Tris-HCl, 40% (w/v) glycerol, 0.03% (w/v) of bromophenol blue and xylene cyanol) at a 3:1 volume ratio for 3 h at 30 °C. The samples were then electrophoresed at 4 °C and 2 W in a 14% polyacrylamide (29:1 acrylamide/bis-acrylamide) gel made in THEM_3_ buffer (34 mM Tris, 66 mM HEPES, 0.1 mM EDTA, and 3 mM MgCl_2_) which is also used as a running buffer.^70^ The gel typically ran for about 14 h with a running length of 15 cm. Gels were stained by SYBR Gold (Invitrogen) in Tris buffer (10 mM Tris, pH 8.0) for 10 min. After staining, images were captured with the CCD camera on the UV transilluminator (SmartView 111, Major Science) and then analyzed with ImageJ software (NIH).

### Preparation and Purification of Biotinyl-4-Cyanotryptophan Conjugated RNA (Holo-Form Sample)

S-1A.1-a (1.3 nmol) was incubated with 1000 μM B4CNWO in the aminoacylation buffer (100 mM HEPES, 100 mM NaCl, 100 mM KCl, 20 mM MgCl_2_, and 20 mM CaCl_2_, pH 8.00) at 4 °C for 60 min. The process was repeated three times to maximize the labeling efficiency. S-2.1-a (1.3 nmol) was incubated with 1000 μM B4CNWO in the aminoacylation buffer at room temperature for 20 min. After the incubation, the unreacted B4CNWO were removed by Bio-Gel *P*6̅ (Bio-Rad) at room temperature with Tris-NaCl buffer as the mobile phase. This process yielded the Biotinyl-4-cyanotryptophan conjugated RNA, which we referred to as the holo-form sample. The RNA concentration of each sample was determined by the absorbance at 260 nm using Biophotometer (Eppendorf).

### Measuring Fluorescence Spectra of Divalent Ion Titration and Varying Temperature Experiments

Fluorescence spectra were acquired using a FluoroMax Plus Spectrofluorometer (Horiba Scientific) with a xenon lamp. Measurements were conducted in 1.0 cm quartz cells with a spectral resolution of 1.0 nm and an integration time of 1.0 s/nm. Samples were excited at 302 nm with an excitation slit width of 10 nm and an emission slit width of 5 nm. Before each measurement, samples were allowed to equilibrate in the cuvette for at least 5 min. In Mg^2+^ and Ca^2+^ titration experiments, the final RNA-B4CNW concentrations were 50 nM for S-2.1-a and 100 nM for S-1A.1-a in Tris-NaCl buffer. To prevent potential RNA degradation, 80 U of RNase inhibitors (RNAok, SMOBIO, Taiwan) were added to the cuvette. One M stock solutions of Mg^2+^ or Ca^2+^ in Tris-NaCl buffer were titrated and mixed into the cuvette, with 5 min of equilibration before each measurement. Emission scans were collected from 390 to 430 nm. For temperature variation experiments, RNA samples and scanning parameters remained consistent with those described above. The measuring temperatures varied from 4 to 74 °C with a resolution of 2 °C per scan. Before each scan, samples were equilibrated at the target temperature for 5 to 10 min.

### Fluorescence Signal Analysis

The average fluorescence wavelength (⟨λ⟩) was calculated using the following equation:

$$\left\langle \lambda \right\rangle =\frac{\underset{\lambda }{\sum }\lambda \times I\left(\lambda \right)}{\underset{\lambda }{\sum }I\left(\lambda \right)}$$

where *I*(λ) is the intensity of wavelength λ. The average fluorescence wavelength (Δ⟨λ⟩) change in different conditions is obtained by

$$\Delta \left\langle \lambda \right\rangle =\langle \lambda \rangle_{i}-{\langle \lambda \rangle }_{0}$$

where ⟨λ⟩_0_ and ⟨λ⟩*_i_* are the average wavelengths of the initial and variable conditions (i.e., temperature and divalent ion concentrations), respectively. The normalized average wavelength change Δ⟨λ⟩*_N_* of different temperatures were calculated by normalizing the maximum wavelength change at the highest temperature to 1. The onset of thermally induced unfolding temperature (T_*x*_) is then determined by the intersection of the two linear regression lines. The RANSAC algorithm was utilized to select the inliers for linear regression within the specific temperature ranges.^103^ This involved randomly selecting two points for each iteration, with 2000 iterations set to ensure a reproducible result. The threshold was set at an average of 0.04 to eliminate outliers. The inliers that exhibit consistent linear behavior comprise more than 75% of the data points. The *R* ^2^ values of the fits were all greater than 0.85.

The relative peak fluorescence intensity change (Δ*F*) was calculated as

$$\Delta F=\frac{F_{max}^{i}-F_{max}^{o}}{F_{max}^{o}}$$

where $F_{max}^{i}$ and $F_{max}^{0}$ are the intensity of the fluorescence peak for variable and initial conditions, respectively.

### AlphaFold3 Prediction of Self-aminoacylating Ribozyme Structures

The predicted ribozyme structures for S-1A.1-a and S-2.1-a were generated using the AlphaFold 3 server (http://alphafoldserver.com/) with default settings. This server produces five structural predictions for each job, which are ranked according to the ranking score metric. For the structure without Mg^2+^, the input consisted exclusively of the ribozyme sequences for S-1A.1-a and S-2.1-a. In the Mg^2+^-bound structures, two seed configurations were employed: one with a single Mg^2+^ and the other containing two Mg^2+^.

## Supplementary Material



## Notes

Quantitative and qualitative analyses of RNA detection and RNA-BYO formation (Figure S1); dependence of ribozyme activity on [Mg^2+^] and [Ca^2+^] (Figure S2); effect of higher [BYO] on S-2.1-a activity (Figure S3); synthesis route, NMR spectra, and MALDI-TOF analysis of B4CNWO (Figures S4–S6); ribozyme activity using B4CNWO as substrate (Figure S7); streptavidin gel-shift and Mg-native PAGE analyses under various ionic conditions (Figures S8–S12); EDTA reversal of Mg^2+^-induced fluorescence shifts, thermal stability, and Ca^2+^ fluorescence effects (Figures S13–S15); two-dimensional illustrations of AlphaFold 3 predicted tertiary structures (Figure S16); synthesis Procedures for B4CNWO precursors; ^1^H NMR data for synthetic precursors (Table S1); and quantification of gel band intensities for global conformational analysis (Table S2) (PDF)
