# Supplementary material for: Decoupling Global and Local Structural Changes in Self-aminoacylating Ribozymes Reveals the Critical Role of Local Structural Dynamics in Ribozyme Activity
Source: JACS Au. 2025 May 9;5(5):2172–85. doi: 10.1021/jacsau.5c00146 (PMC12117395; doi:10.1021/jacsau.5c00146)
Supplement: Supplementary file 1 [file au5c00146_si_001.pdf]

Supplementary Materials for

Decoupling Global and Local Structural Changes in Self-aminoacylating Ribozymes Reveals the Critical Role of Local Structural Dynamics in Ribozyme Activity

Yu-Kai Cheng, Hsing-Hui Chu, Ning-Jun Yang, and Yei-Chen Lai*

Department of Chemistry, National Chung Hsing University 145 Xingda Rd., South Dist, Taichung City 402202, Taiwan

**Corresponding email: yeichenlai@nchu.edu.tw*

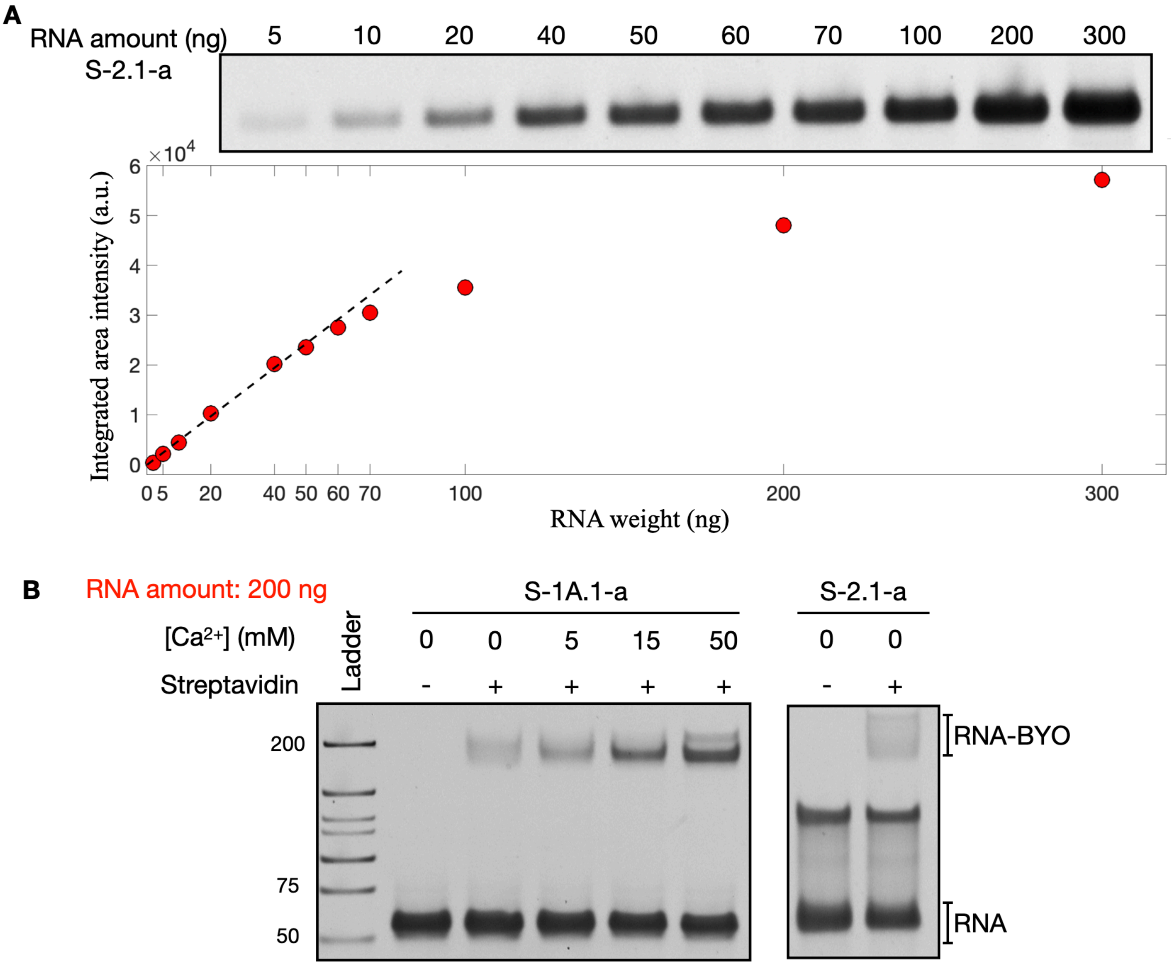

Figure S1. Quantitative and qualitative analysis of RNA detection and RNA-BYO formation. (A) The relationship between RNA weight and integrated area intensity for S-2.1-a. RNA samples ranging from 5 to 300 ng were analyzed via gel electrophoresis, and the integrated area intensities were plotted against RNA amount. A linear fit (dashed line) was applied to data in the 5–70 ng range. **(B)** The qualitative assay used 200 ng of RNA, outside the linear detection range, to evaluate the effect of [Ca^{2+}] on RNA-BYO formation. The presence or absence of streptavidin and varying [Ca^{2+}] (0–50 mM) were assessed. RNA and RNA-BYO bands are indicated.

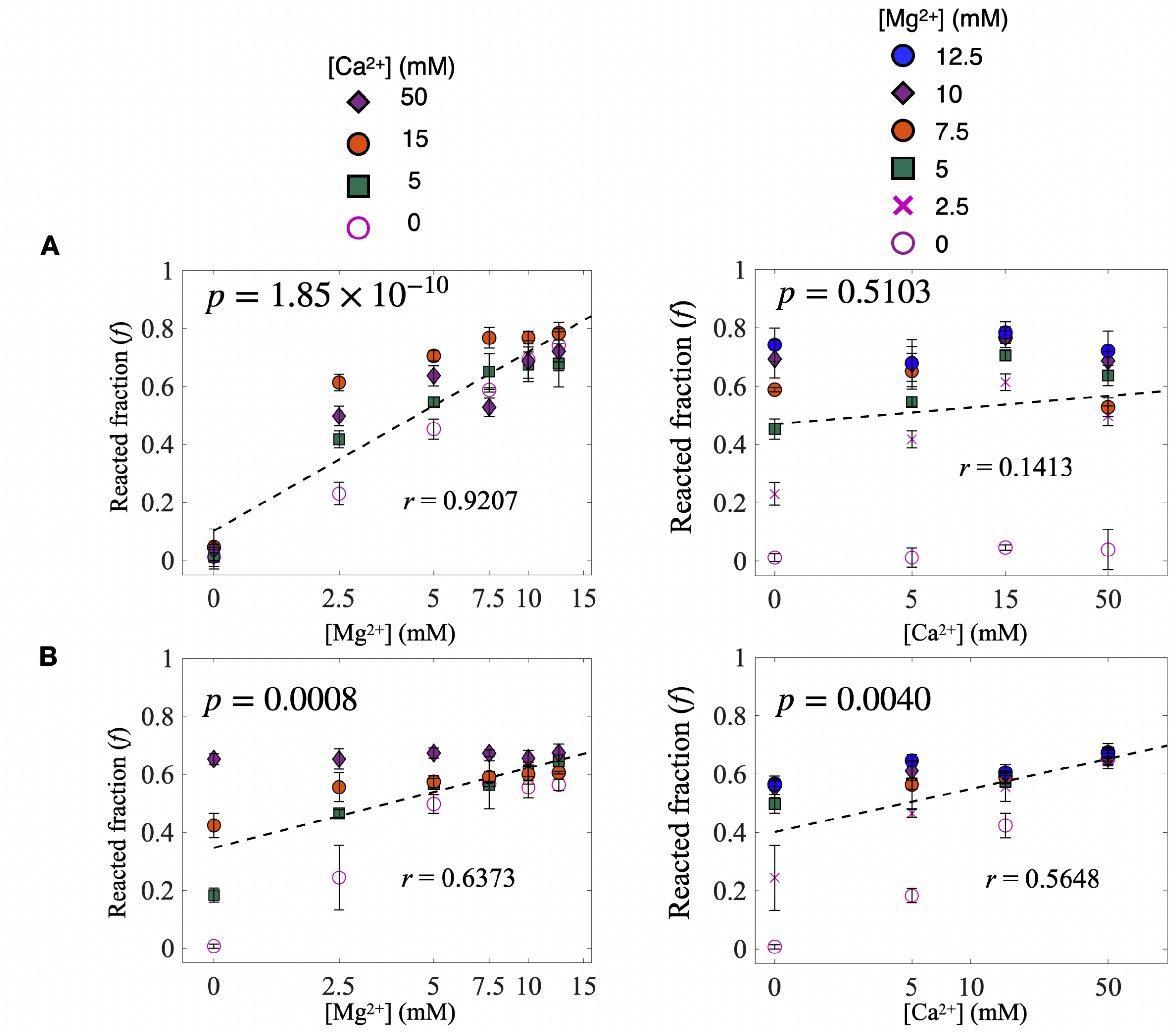

Figure S2. The dependence of S-1A.1-a and S-2.1-a activity on [Mg^{2+}] and [Ca^{2+}]. (A) The activity relationship of S-1A.1-a to [Mg^{2+}] (left) and [Ca^{2+}] (right). **(B)** The activity relationship of S-2.1-a to [Mg^{2+}] (left) and [Ca^{2+}] (right). The experimental conditions are consistent with those used in Figure 2, with [BYO] set at 500 μM for S-1A.1-a and 20 μM for S-2.1-a. The best linear fits (dashed line) are displayed. The calculated Pearson coefficients (*r*) and the *p*-value are shown in the figures. The error bars represent the standard deviation of the mean (n = 3).

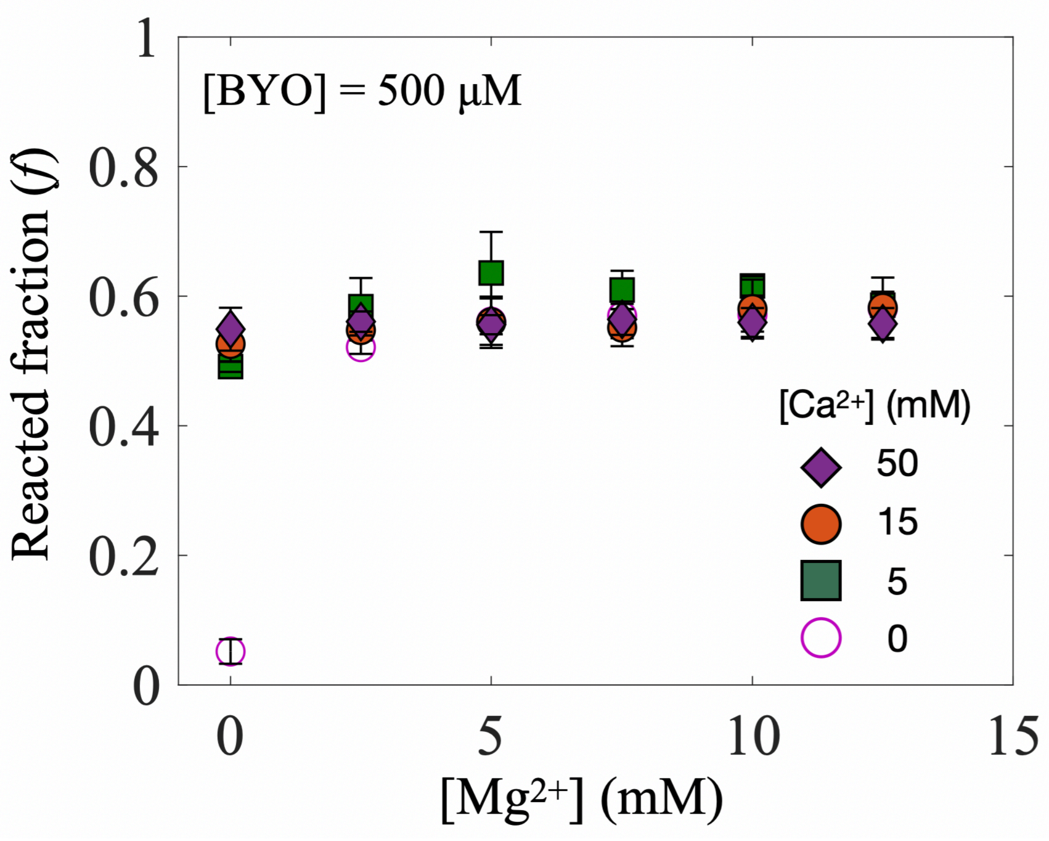

Figure S3. Dependence of S-2.1-a activity on [Mg^{2+}] and [Ca^{2+}] at higher [BYO]. The activity relationship of S-2.1-a with [BYO] = 500 µM as a function of [Mg^{2+}] at different [Ca^{2+}] (0, 5, 15, 50 mM). The result shows that the fast catalytic rate of S-2.1-a at high substrate concentration reduces the difference between the different divalent ion conditions. The error bars represent the standard deviation of the mean (n = 3).

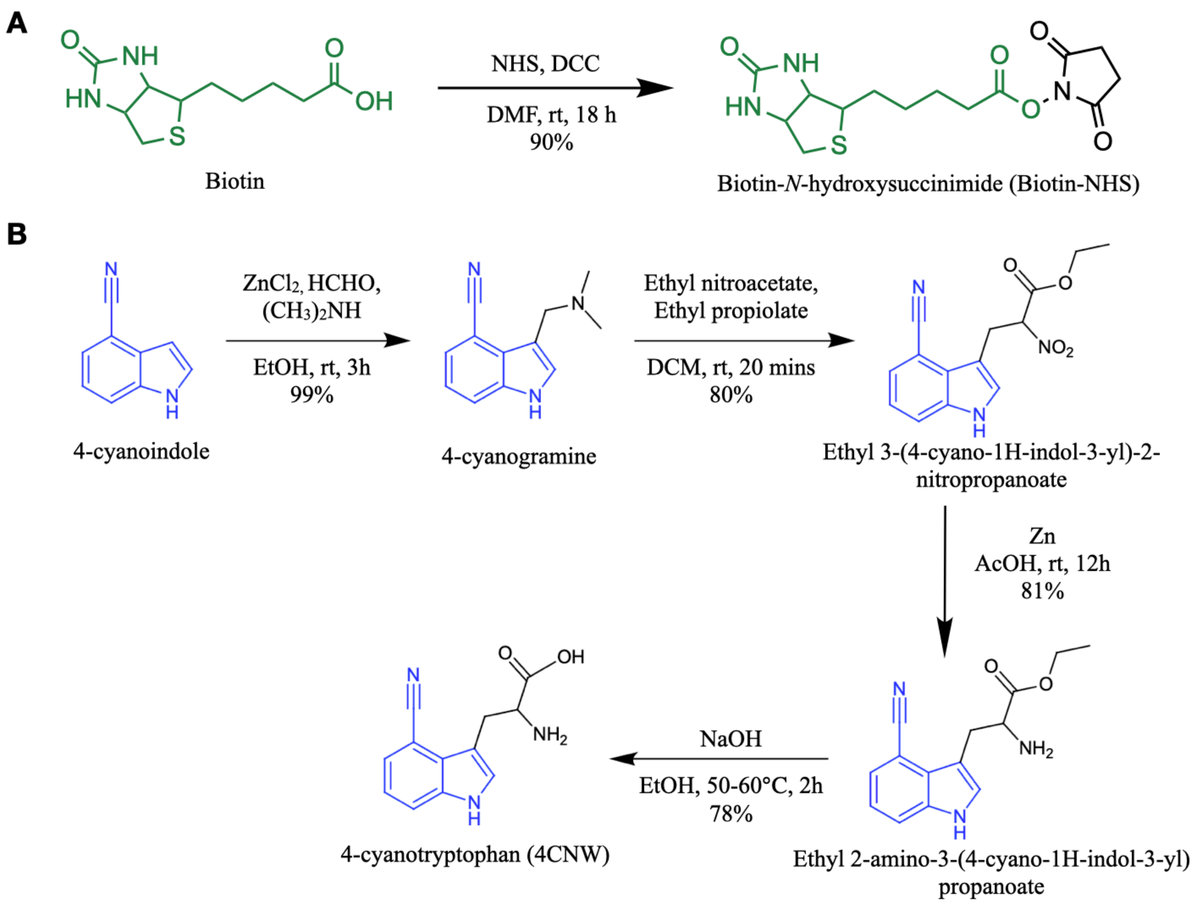

Figure S4. Synthesis route of precursors for Biotinyl-4-cyanotryptophan-5(4*H*)-oxazolone. (A) Synthesis of Biotin-hydroxysuccinimide (Biotin-NHS). **(B)** Synthesis of 4-cyanotryptophan (4CNW). The ^{1}H-NMR peaks of all compounds are listed in Table S1.

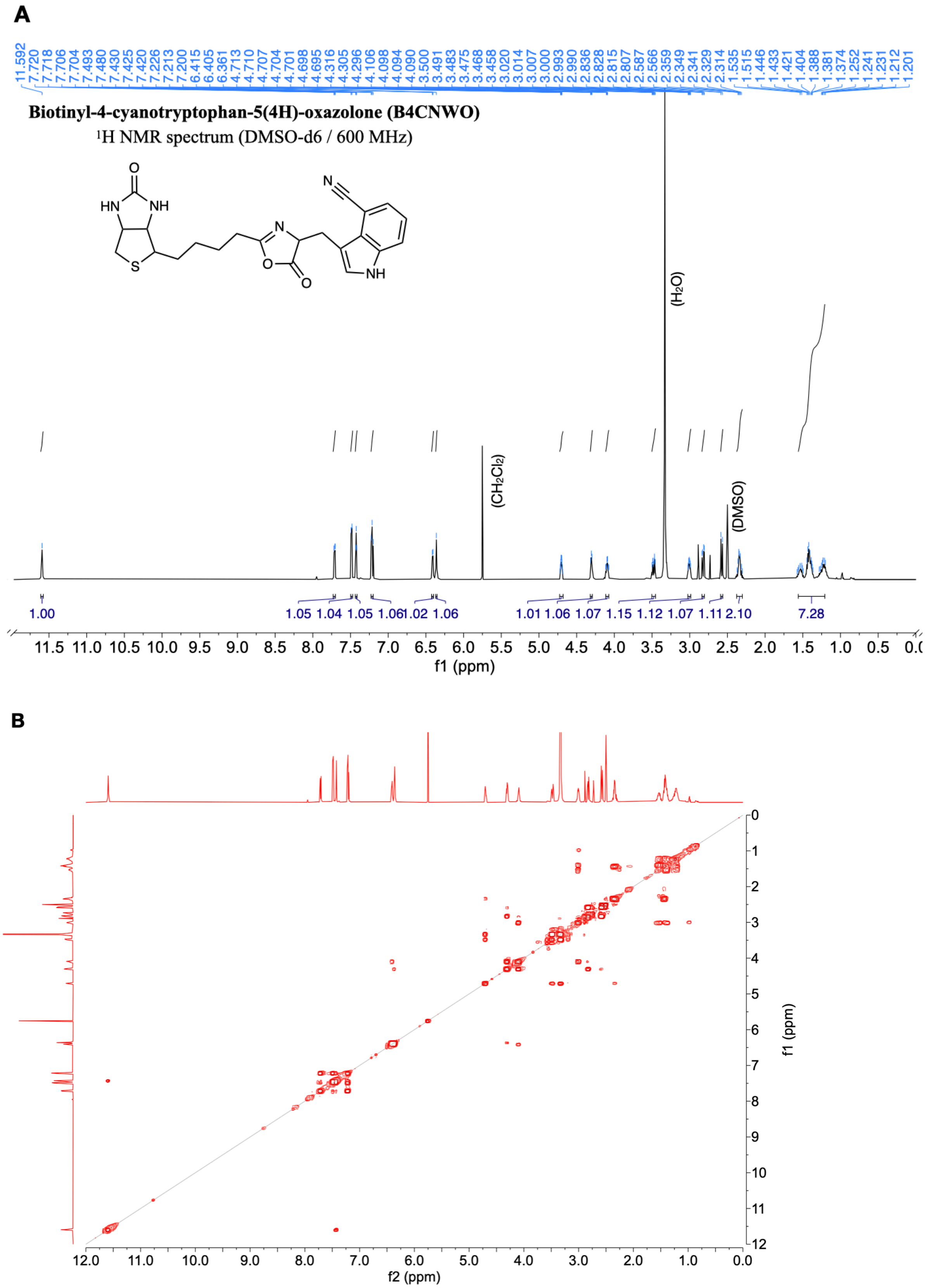

Figure S5. NMR spectra of biotinyl-4-cyanotryptophan-5(4*H*)-oxazolone. The NMR spectra were recorded on an Agilent DD2 (600 MHz). The spectra shown are (**A**) ^{1}H, (**B**)2D-COSY, and (**C**) ^{13}C.

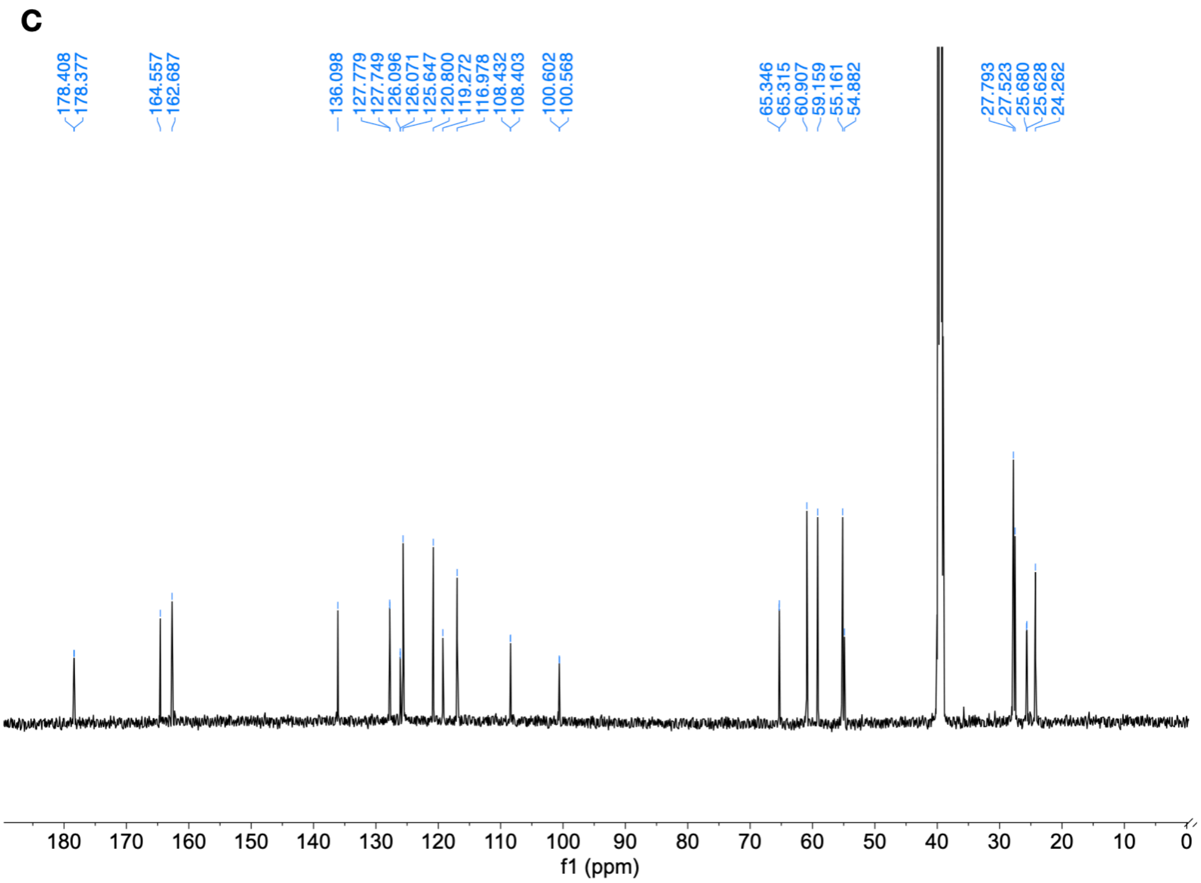

Figure S5. continued.

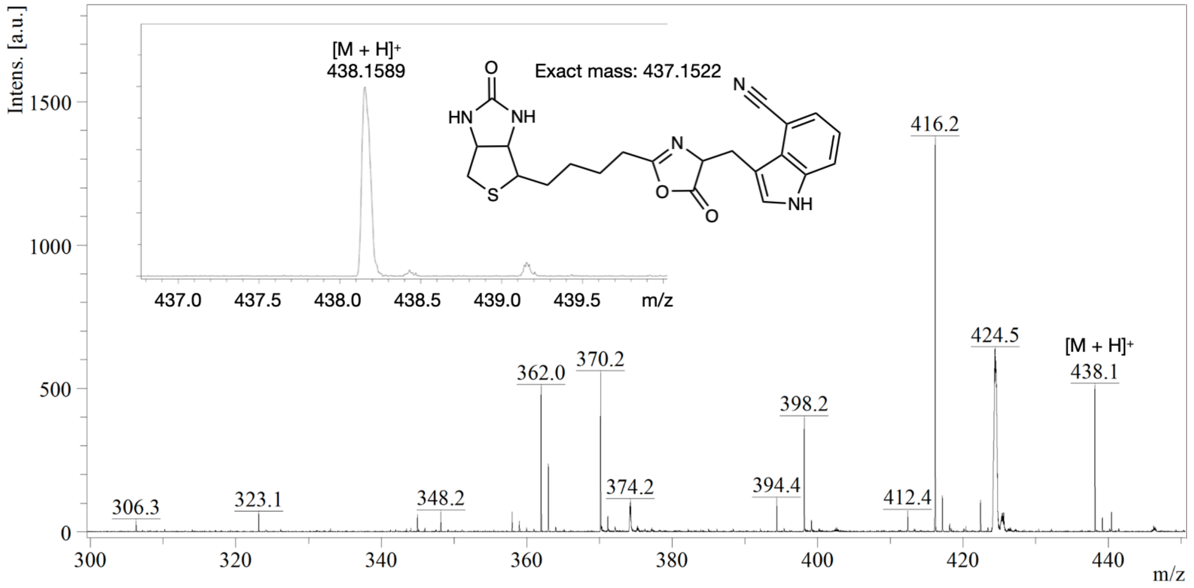

Figure S6. MALDI-TOF mass spectrum of biotinyl-4-cyanotryptophan-5(4*H*)-oxazolone. The spectrum was obtained using a Bruker Daltonics UltrafleXtreme MALDI-TOF/TOF mass spectrometer with an Nd: YAG laser (λ = 355 nm) controlled by FlexControl data collection software. The measurement error is 1.1899 ppm.

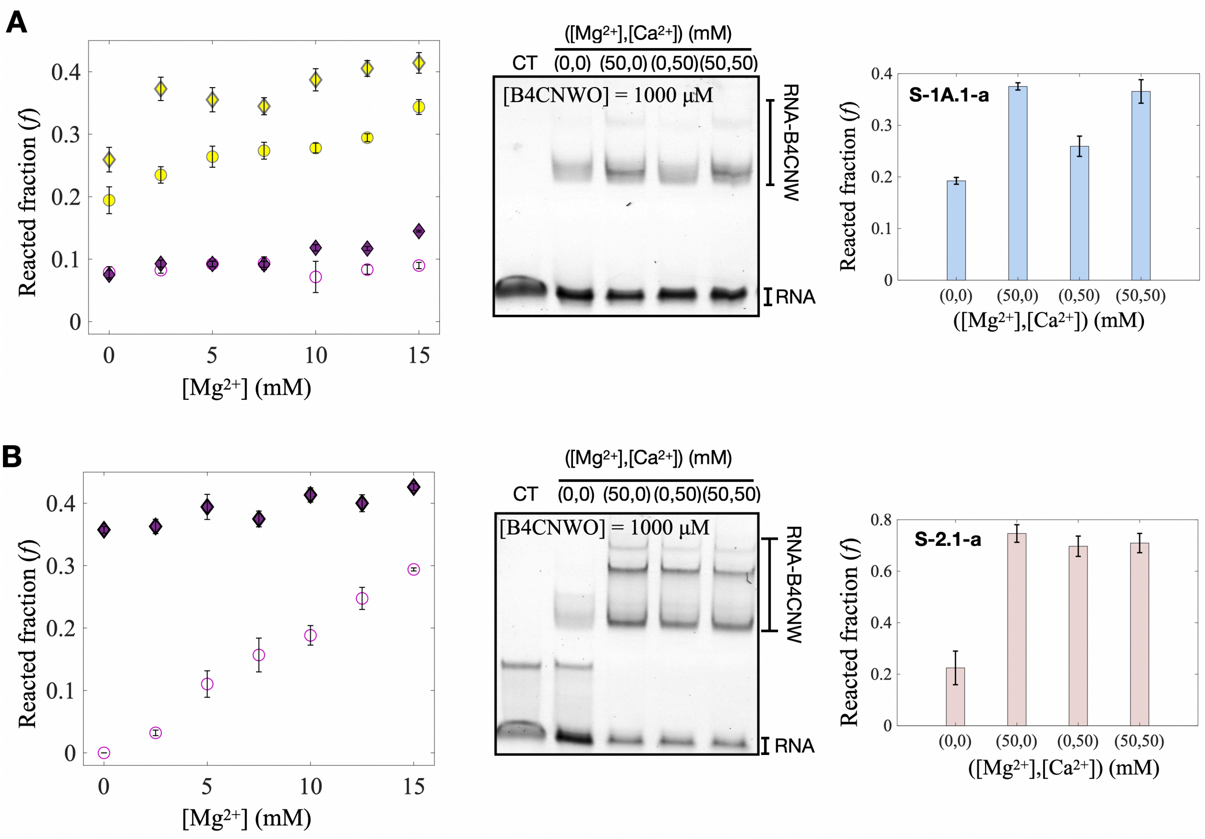

Figure S7. S-1A.1-a and S-2.1-a activity using B4CNWO as a substrate under varying [Mg^{2+}] and [Ca^{2+}] conditions. (A) Results for S-1A.1-a (B) results for S-2.1-a. The left-most panels show the reacted fraction (*f*) for the two ribozymes using [B4CNWO] = 1000 µM (yellow) and 500 µM (purple) for S-1A.1-a, and 20 µM (purple) for S-2.1-a. Reactions were incubated at room temperature for 100 min under 0 mM Ca^{2+} (circles) or 50 mM Ca^{2+} (diamonds). Error bars represent the standard deviation (n = 3). For S-1A.1-a, two substrate concentrations are shown. The 500 µM condition allows a direct comparison with the BYO data in Figure 2, while the 1000 µM condition provides a better signal due to the generally lower reactivity of B4CNWO with this ribozyme. The overall trends are consistent with those observed in Figure 2. Specifically, S-1A.1-a shows greater dependence on Mg^{2+} concentration, whereas S-2.1-a maintains substantial activity in the presence of either Mg^{2+} or Ca^{2+}. However, both ribozymes display lower overall activity with B4CNWO compared to BYO. To further confirm these results, we performed the assay under four defined ionic conditions: (0,0), (50,0), (0,50), and (50,50) mM of [Mg^{2+}] and [Ca^{2+}], respectively. All reactions were incubated in [B4CNWO] = 1000 µM at RT for 100 min (middle gel). The reacted fraction (*f*) is quantified in the right bar graph. S-1A.1-a shows higher reactivity in conditions containing [Mg^{2+}] (50,0 or 50,50) compared to the activity in the Ca^{2+}-only condition (0,50). In contrast, S-2.1-a maintains efficient activity across all ionic conditions, demonstrating a broader tolerance for divalent ions. The right-most bar plots quantify the reacted fractions for each condition. Error bars represent the standard deviation (n = 4).

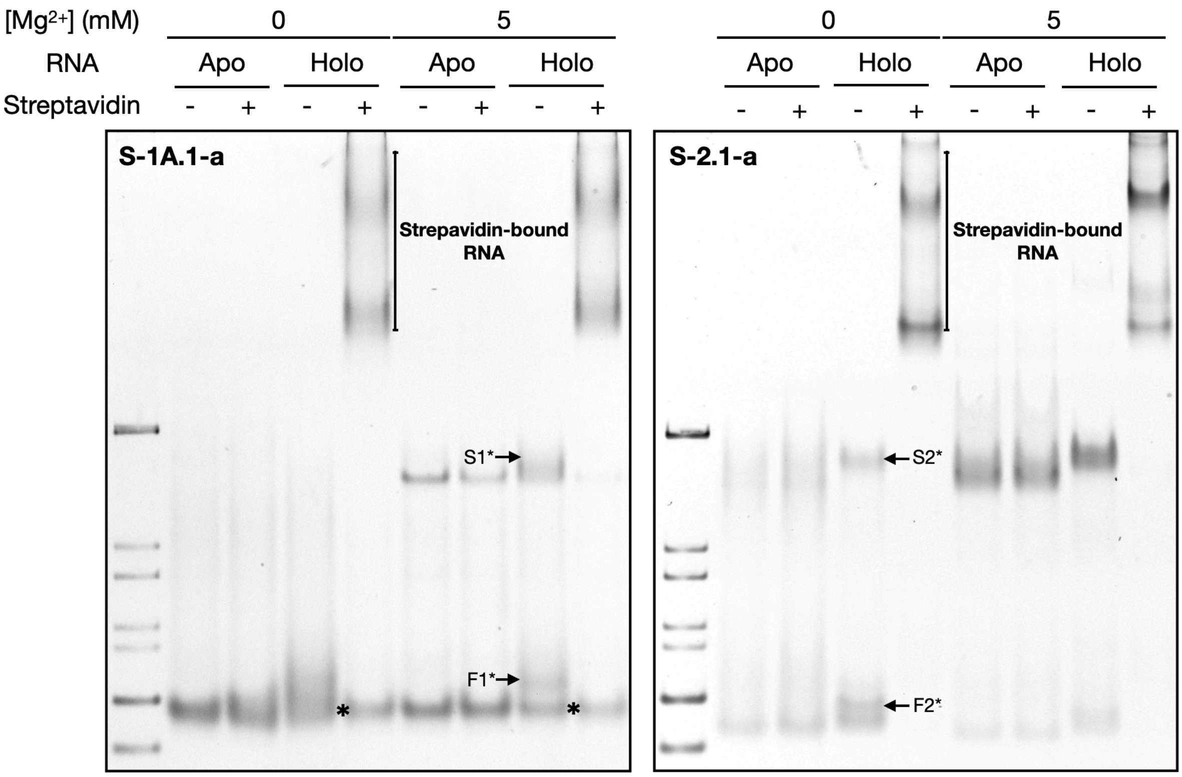

Figure S8. Characterization of bands identified on Mg-native PAGE by streptavidin gel-shift assay. Mg-native PAGE analysis was conducted on S-1A.1-a (left) and S-2.1-a (right) ribozymes at 0 and 5 mM Mg^{2+}. The unconjugated (apo) and B4CNW-conjugated (holo) samples of both ribozymes were analyzed. Samples were incubated with streptavidin to verify B4CNW conjugation. For S-1A.1-a, the F1 band was observed in the apo-form sample without Mg^{2+}, while the holo-form sample showed a band labeled F1*. The asterisk in the gel denotes unreacted F1 in the holo-form sample, indicating a mixture of unconjugated and conjugated RNA. For S-2.1-a, the holo-form sample displayed two bands labeled F2* and S2*. Streptavidin incubation resulted in significant upward shifts for the RNA bands, regardless of their conformation. These results demonstrate successful B4CNW conjugation in ribozymes and consistent structural transitions from compact to extended forms with rising Mg^{2+} concentration, as explained in the main text.

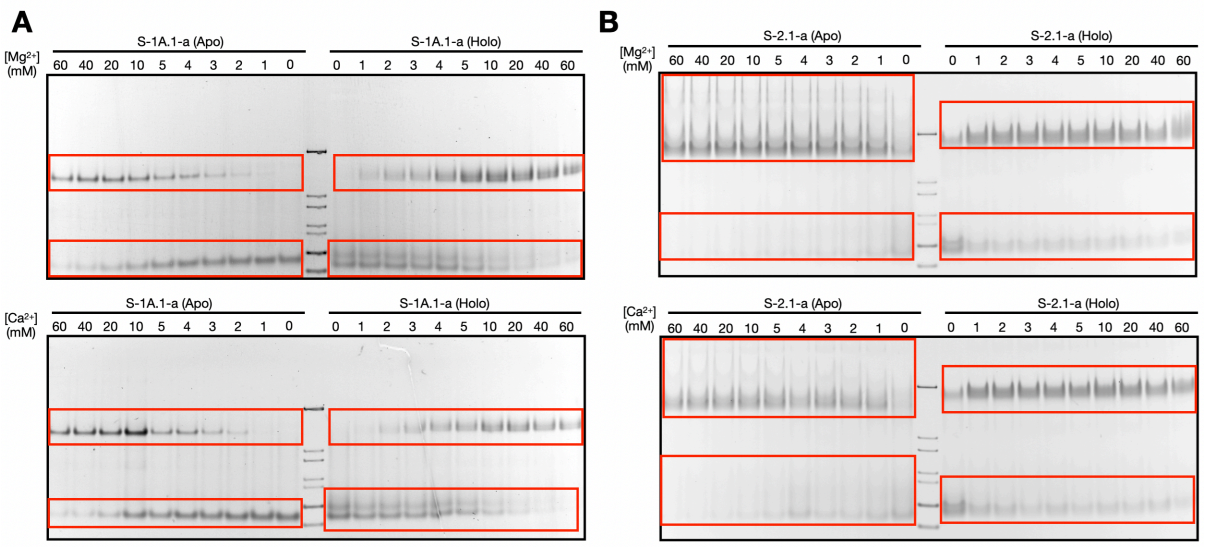

Figure S9. Mg-native PAGE analysis of S-1A.1-a and S-2.1-a ribozyme conformations at varying Mg^{2+} and Ca^{2+} concentrations. Mg-native PAGE analysis of (A) S-1A.1-a and (B) S-2.1-a ribozymes, including both apo-form and holo-form samples, at different Mg^{2+} (top) and Ca^{2+} (bottom) concentrations ranging from 0 mM to 60 mM. The results showed that for both ribozymes, Mg^{2+} and Ca^{2+} similarly shift the structural equilibrium from compact to extended structures. The red-highlighted areas indicate the specific regions used to quantify slow and fast migrating bands by ImageJ (Table S2).

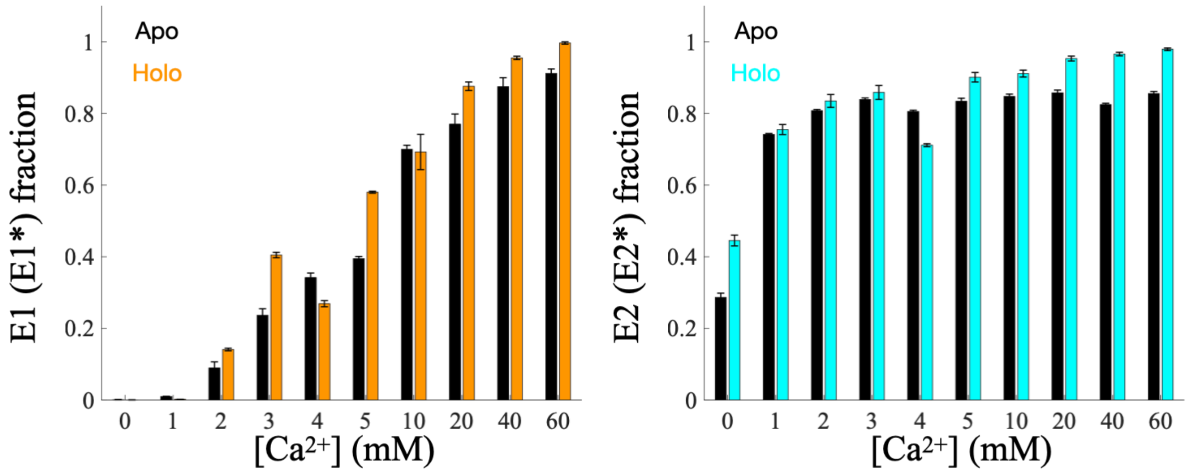

Figure S10. Quantitative analysis of extended conformations shown in Mg-native PAGE for S-1A.1-a and S-2.1-a at varying Ca^{2+} concentrations. The analysis of the fraction of extended conformations (E1 and E1* for S-1A.1-a, E2 and E2* for S-2.1-a) at varying Ca^{2+} concentrations (0 to 60 mM). The left panel shows the fraction of E1 (black) and E1* (orange) for S-1A.1-a, and the right panel shows the fraction of E2 (black) and E2* (cyan) for S-2.1-a. The error bars represent the standard deviation of the mean (n = 3).

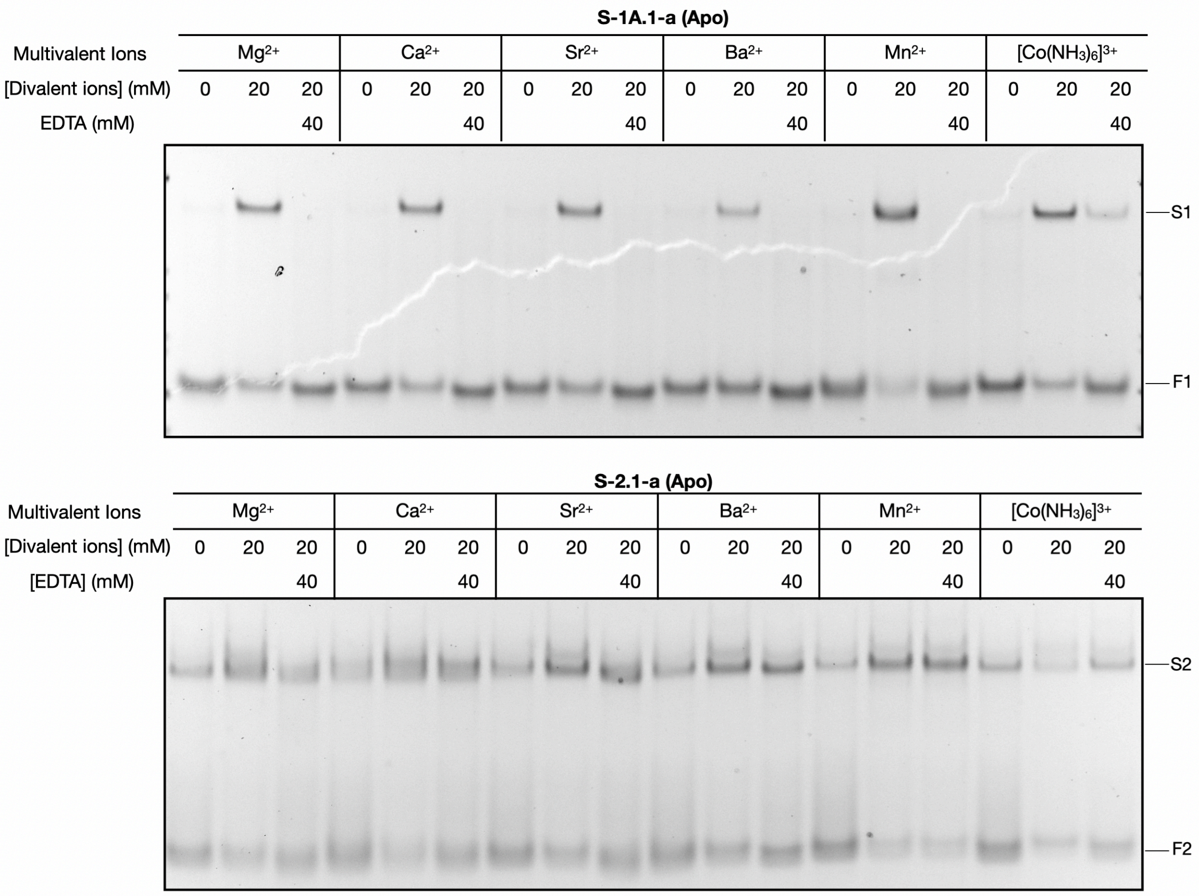

Figure S11. Mg-native Page analysis of S-1A.1-a and S-2.1-a under varying multivalent ion conditions. The global structural equilibrium of S-1A.1-a (top) and S-2.1-a (bottom) ribozymes were analyzed in the presence of different divalent ions, including Mg^{2+}, Ca^{2+}, Sr^{2+}, Ba^{2+}, Mn^{2+}, and $[Co(NH_3)_6]^{3+}$, at 0 or 20 mM concentrations. The gel bands reveal structural changes and bent conformations induced by each ion, as reflected in the differences between the S1/S2 and F1/F2 bands. The addition of 40 mM EDTA chelated the multivalent ions, demonstrating that the conformational changes are primarily driven by RNA interactions with the multivalent ions.

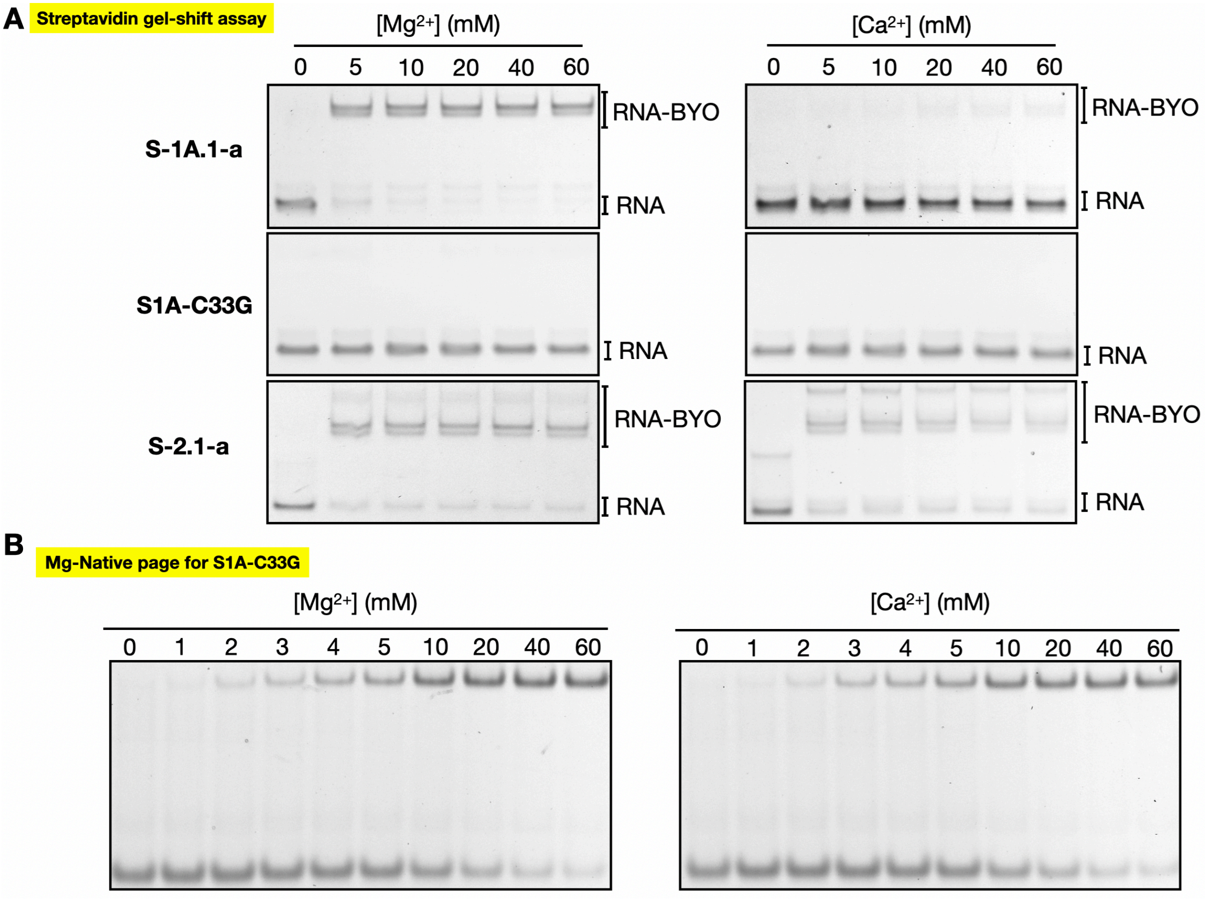

Figure S12. Streptavidin gel-shift assay and Mg-native PAGE analysis of S-1A.1-a, S1A-C33G, and S-2.1-a under high Mg^{2+} and Ca^{2+} concentrations. (A) Streptavidin gel-shift assay of S-1A.1-a, S1A-C33G, and S-2.1-a at high Mg^{2+} and Ca^{2+}, concentrations (0 to 60 mM). The ribozymes were incubated with [BYO] = 500 µM for 90 min. The left panels display results under varying Mg^{2+} concentrations. The right panels show results under varying Ca^{2+} concentrations. S-2.1-a exhibits reactivity in buffers containing either Mg^{2+} or Ca^{2+}, whereas S-1A.1-a shows very low reactivity in buffers containing only Ca^{2+}. The mutant S1A-C33G shows almost no activity in both Mg^{2+} and Ca^{2+} conditions. (B) Mg-native PAGE analysis of S1A-C33G under varying Mg^{2+} and Ca^{2+} concentrations (0 to 60 mM). The global conformation equilibrium of the mutant shifts as observed in its wild-type sequence, S-1A.1-a; however, S1A-C33G shows almost no catalytic activity.

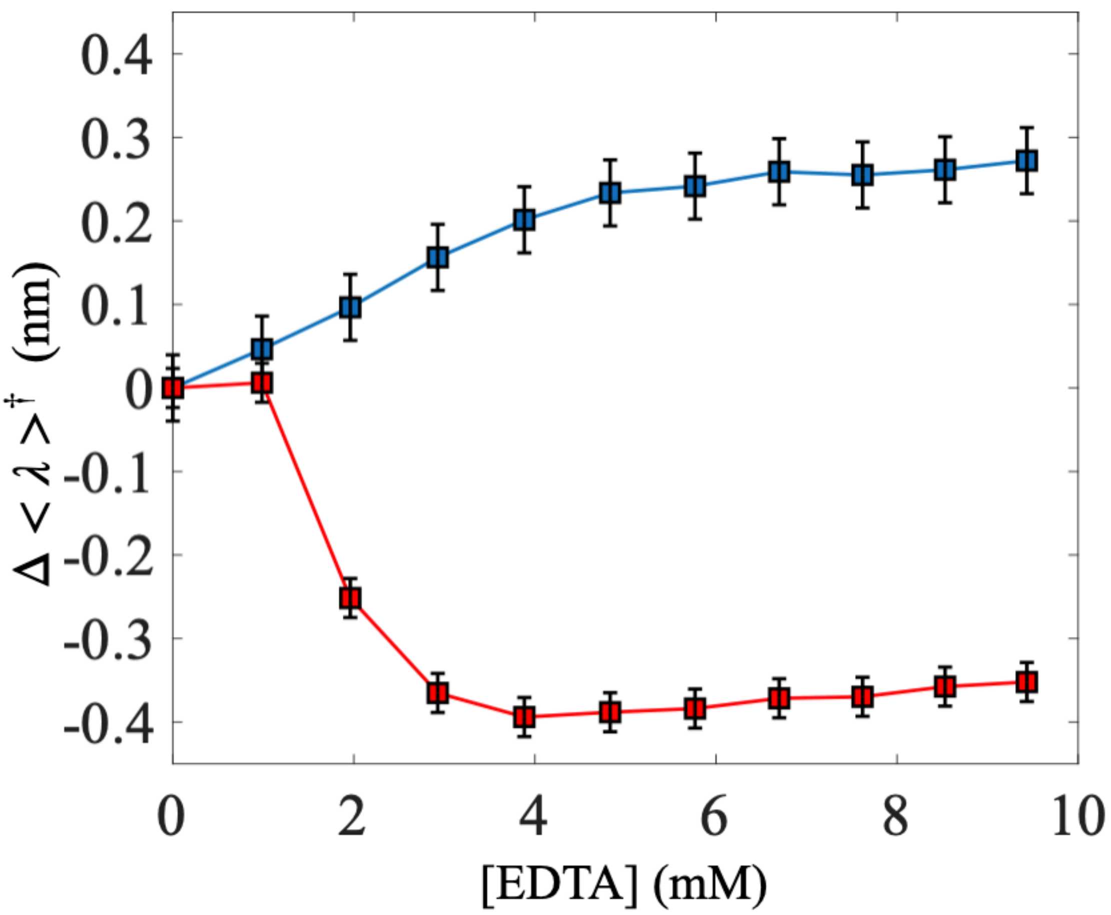

Figure S13. Reversal of Mg^{2+}-induced fluorescence wavelength changes by EDTA titration. The change in average fluorescence wavelength ($\Delta\!<\lambda>^{\dagger}$) was calculated as the difference between $<\lambda>$ at a given [EDTA] and $<\lambda>$ at [EDTA] = 0 mM (baseline condition with 10 mM Mg^{2+}). $\Delta\!<\lambda>^{\dagger}$ values are shown for B4CNW-conjugated S-1A.1-a (blue) and S-2.1-a (red) at 25 °C. Error bars represent the standard deviation (n = 5).

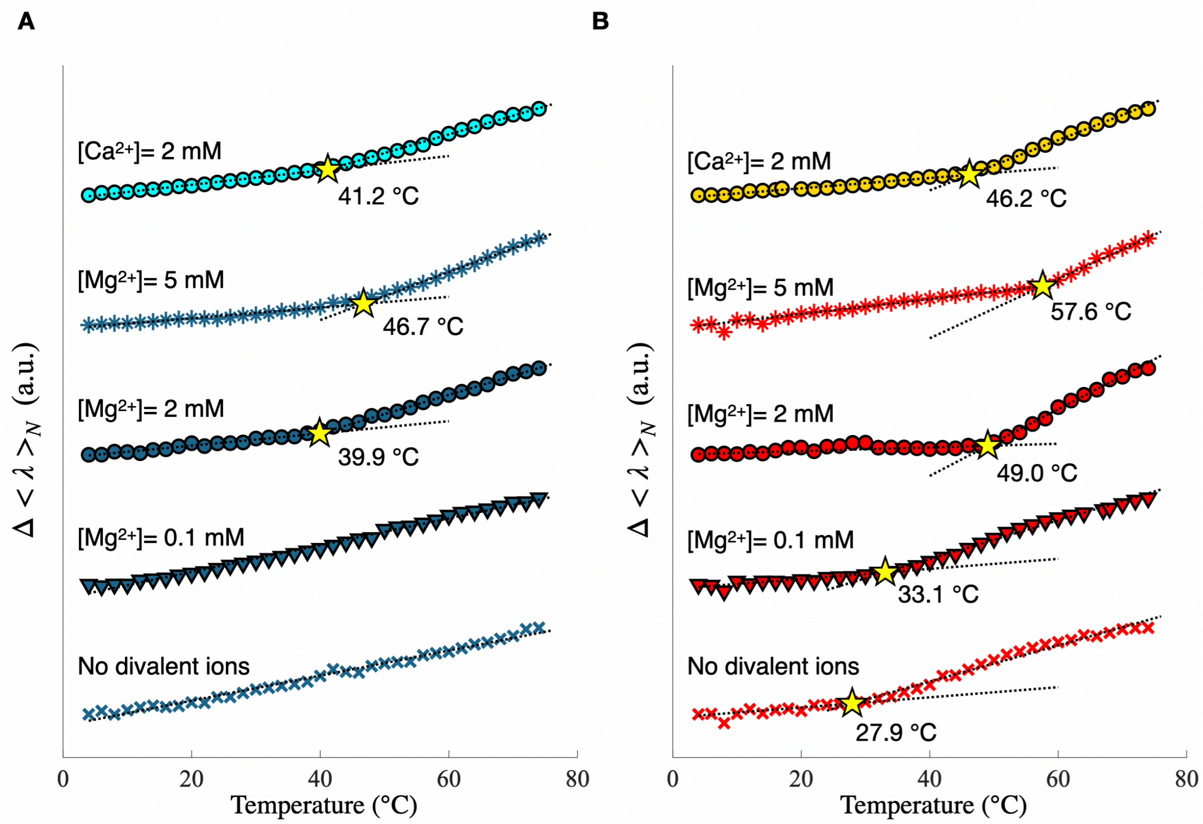

Figure S14. The thermal stability of ribozyme structures is affected by varying concentrations of divalent ions. Normalized average fluorescence wavelength change ($\Delta\langle\lambda\rangle_N$) as a function of temperature for (**A**) S-1A.1-a and (**B**) S-2.1-a under different ion conditions: 0 mM divalent ions (red and blue crosses), 0.1 mM Mg^{2+} (red and blue triangles), 2 mM Mg^{2+} (red and blue circles), 5 mM Mg^{2+} (red and blue asterisks), and 2 mM Ca^{2+} (yellow and cyan circles). Yellow stars mark the onset temperatures (T_x), determined as described in Methods. The determining T_x values are shown.

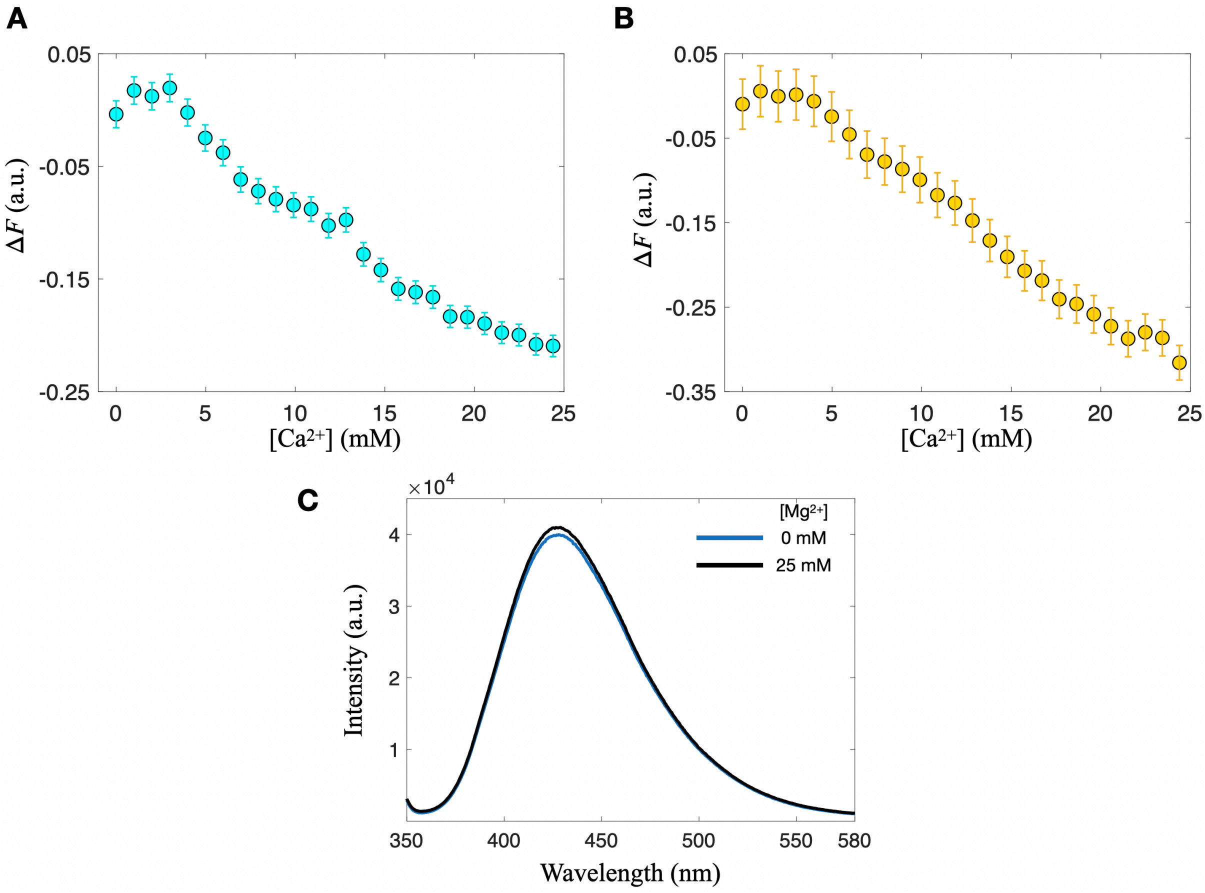

Figure S15. Effect of Ca^{2+} on fluorescence intensity and the control experiments with free B4CNW. The relative peak fluorescence intensity (ΔF) during the titration of [Ca^{2+}] for at 25 °C for (**A**) S-1A.1-a and **(B)** S-2.1-a. The error bars represent the standard deviation of the mean (n = 5). (C) Fluorescence emission spectra of free B4CNW in the absence (0 mM) and presence (25 mM) of Mg^{2+}.

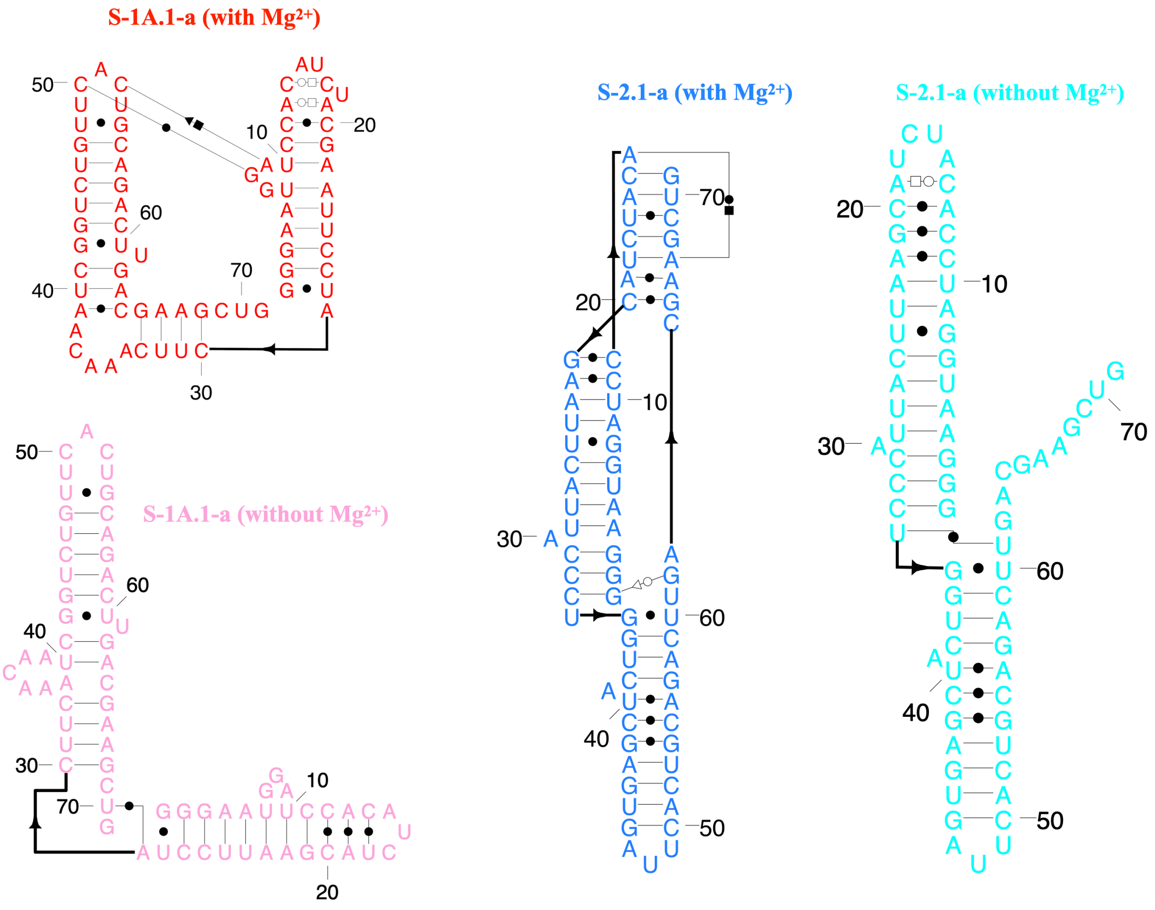

Figure S16. Two-dimensional illustrations of Alphafold 3 predicted tertiary structures. Two-dimensional drawings of Alphafold 3 predicted tertiary structures of S-1A.1-a and S-2.1-a in the presence (red and blue) or absence (pink and cyan) of Mg^{2+}.

Synthesis Procedures for Precursors of Biotinyl-4-cyanotryptophan-5(4*H*)-oxazolone (B4CNWO)

4-cyanogramine

To a 25 mL round-bottom flask containing a solution of 4-cyanoindole (0.2843 g, 2 mmol) and $ZnCl_2$ (0.4088 g, 3 mmol) in 5 mL of EtOH, chilled to 4°C, was slowly added formaldehyde (163 µL of 37% aqueous solution) and dimethylamine (1 mL of 2 M solution in THF). The reaction mixture was stirred at room temperature for 3 h. The contents were then made basic with 1 mL of 20% aqueous sodium hydroxide solution and extracted three times with 10 mL of ethyl acetate. The combined ethyl acetate extracts were dried over anhydrous magnesium sulfate, filtered, and the solvent was removed under reduced pressure. The resulting residue was dissolved in 8 mL of water, acidified with 1 mL of 20% HCl, and filtered. Finally, 20% NaOH was slowly added to obtain the title compound as a white solid (99%).

Ethyl 3-(4-cyano-1H-indol-3-yl)-2-nitropropanoate

To a suspension of 4-cyanogramine (0.3982 g, 2 mmol) in 10 mL of dichloromethane was added ethyl nitroacetate (222 µL) in a single portion followed by ethyl propiolate (203 µL). The reaction mixture was poured into a separatory funnel containing ethyl acetate (20 mL). The organic solution was washed with 1 M HCl (15 mL), water (15 mL), and brine (15 mL) once each. The organic layer was dried over anhydrous magnesium sulfate, and the volatiles were removed under reduced pressure to obtain the title compound as a yellow solid (80%).

Ethyl 2-amino-3-(4-cyano-1H-indol-3-yl) propanoate

To a solution of ethyl 3-(4-cyano-1H-indol-3-yl)-2-nitropropanoate (0.2871 g, 1 mmol) in 10 mL of acetic acid was added powdered zinc (1.046 g, 16 mmol) portion-wise, with 4 mmol per portion added every 20 min. The mixture was refluxed and stirred overnight at room temperature. The excess zinc was then filtered off, and the acetic acid was removed under reduced pressure. The residue was dissolved in 0.3 M HCl and extracted twice with dichloromethane. The aqueous layer was adjusted to pH 7-8 with 28% ammonia solution and extracted again with dichloromethane. The combined organic extracts were dried over anhydrous magnesium sulfate and concentrated to yield the bright oily yellow title compound (81%).

4-cyanotryptophan (4CNW)

To a solution of ethyl 2-amino-3-(4-cyano-1H-indol-3-yl)propanoate (0.2571 g, 1 mmol) in 5 mL of EtOH in a 25 mL round-bottom flask was added 1 M NaOH (2 mL). The mixture was stirred and refluxed at 50°C for 2 h. The solvent was removed under reduced pressure, and the product was precipitated by adding water and acidifying with 1 M HCl. The precipitate was centrifuged, the liquid was removed via pipette, and the solid was dried under a desiccator to obtain the title compound (78%).

Biotinyl-*N*-hydroxysuccinimide

To a solution of D-biotin (0.4886 g, 2 mmol) in 10 mL of DMF in a 25 mL round-bottom flask was added N-hydroxysuccinimide (0.2302 g, 2 mmol) and N,N'-dicyclohexylcarbodiimide (0.5158 g, 2 mmol). The mixture was stirred at room temperature overnight. The white precipitate was filtered off, and the filtrate was evaporated. The residue was ground with ether and decanted to obtain the title compound as a white solid (90%).

Table S1 ^{1}H NMR spectra for precursors used in this study

**Compound**	**^{1}H NMR spectrum**
4-cyanogramine	^{1}H NMR (400 MHz, CD_3OD) δ 7.68 (dd, *J* = 8.2, 1.1 Hz, 1H), 7.48 – 7.44 (m, 2H), 7.26 – 7.20 (m, 1H), 3.86 (s, 2H), 2.32 (d, *J* = 1.0 Hz, 6H).
Ethyl 3-(4-cyano-1H-indol-3-yl)-2-nitropropanoate	^{1}H NMR (400 MHz, $CDCl_3$) δ 7.63 (d, *J* = 8.2 Hz, 1H), 7.54 (d, *J* = 7.3 Hz, 1H), 7.30 (d, *J* = 8.6 Hz, 2H), 7.26 (s, 1H), 5.67 (dd, *J* = 10.8, 4.7 Hz, 1H), 4.34 (q, *J* = 7.2 Hz, 2H), 4.04 (dd, *J* = 15.5, 4.7 Hz, 1H), 3.86 (dd, *J* = 15.5, 10.8 Hz, 1H), 1.32 (t, *J* = 7.1 Hz, 3H).
Ethyl 2-amino-3-(4-cyano-1H-indol-3-yl) propanoate	^{1}H NMR (400 MHz, DMSO-d6) δ 7.71 – 7.67 (m, 1H), 7.49 – 7.45 (m, 1H), 7.42 (s, 1H), 7.19 (dd, *J* = 8.2, 7.3 Hz, 1H), 3.97 (qd, *J* = 7.1, 1.1 Hz, 2H), 3.69 – 3.62 (m, 1H), 3.20 (ddd, *J* = 14.5, 6.7, 0.8 Hz, 1H), 3.09 (ddd, *J* = 14.5, 7.4, 0.8 Hz, 1H), 1.04 (t, *J* = 7.1 Hz, 3H).
4-cyanotryptophan (4CN)	^{1}H NMR (400 MHz, D_2O) δ 7.66 (dd, *J* = 8.2, 1.0 Hz, 1H), 7.44 (dd, *J* = 7.4, 1.0 Hz, 1H), 7.28 (s, 1H), 7.21 – 7.14 (m, 1H), 3.54 (t, *J* = 7.1 Hz, 1H), 3.17 (dd, *J* = 14.6, 6.8 Hz, 1H), 3.04 (dd, *J* = 14.6, 7.3 Hz, 1H).
Biotinyl-N-hydroxysuccinimide	^{1}H NMR (400 MHz, DMSO-d6) δ 6.45 – 6.34 (m, 2H), 4.30 (ddt, *J* = 7.5, 5.1, 1.1 Hz, 1H), 4.14 (ddd, *J* = 7.8, 4.4, 1.8 Hz, 1H), 3.10 (ddd, *J* = 8.2, 6.4, 4.3 Hz, 1H), 2.86 – 2.78 (m, 5H), 2.71 – 2.64 (m, 2H), 2.58 (d, *J* = 12.4 Hz, 1H), 1.81 – 1.46 (m, 7H).

Biotinyl-4-cyanotryptophan (B4CNW)	^{1}H NMR (400 MHz, DMSO-d6) δ 11.55 (d, *J* = 3.8 Hz, 1H), 8.16 (dd, *J* = 8.1, 4.6 Hz, 1H), 7.69 (dt, *J* = 8.2, 1.1 Hz, 1H), 7.47 (dd, *J* = 7.4, 0.9 Hz, 1H), 7.36 (d, *J* = 2.5 Hz, 1H), 7.20 (dd, *J* = 8.2, 7.4 Hz, 1H), 4.61 (ddd, *J* = 10.0, 8.1, 5.0 Hz, 1H), 4.27 (dd, *J* = 7.6, 5.1 Hz, 1H), 4.09 – 4.02 (m, 1H), 3.17 (dd, *J* = 15.2, 10.1 Hz, 1H), 3.03 – 2.93 (m, 1H), 2.79 (ddd, *J* = 12.4, 5.1, 2.6 Hz, 1H), 2.55 (d, *J* = 12.4 Hz, 1H), 2.04 (dd, *J* = 8.7, 6.0 Hz, 2H), 1.48 (dt, *J* = 56.1, 7.3 Hz, 5H), 1.18 (td, *J* = 14.3, 7.1 Hz, 3H).
Biotinyl-4-cyanotryptophan-5(4*H*)-oxazolone (B4CNWO)	^{1}H NMR (600 MHz, DMSO-d6) δ 11.59 (s, 1H), 7.71 (dd, J = 8.2, 1.1 Hz, 1H), 7.49 (d, J = 7.4 Hz, 1H), 7.42 (t, J = 3.0 Hz, 1H), 7.21 (t, J = 7.8 Hz, 1H), 6.41 (d, J = 5.7 Hz, 1H), 6.36 (s, 1H), 4.70 (dt, J = 3.8, 1.8 Hz, 1H), 4.31 (t, J = 5.9 Hz, 1H), 4.11 – 4.07 (m, 1H), 3.50 – 3.45 (m, 1H), 3.03 – 2.99 (m, 1H), 2.82 (dd, J = 12.5, 5.1 Hz, 1H), 2.58 (d, J = 12.5 Hz, 1H), 2.38 – 2.30 (m, 2H), 1.56 – 1.21 (m, 7H).

Table S2 Quantification of S1 and F1 band intensities from Figures 4 and S9

$[Mg^{2+}]$ (mM)	S-1A.1-a (Apo form)			S-1A.1-a (Holo form)		
	S1	F1	S1 Fraction	S1*	F1*	S1* Fraction
0	6.45 ± 3.53	6205.26 ± 106.60	0.01 ± 0.01	11.97 ± 12.91	8690.83 ± 350.92	0.01 ± 0.01
1	43.92 ± 45.19	5996.12 ± 196.61	0.01 ± 0.01	614.24 ± 217.97	7035.63 ± 413.90	0.08 ± 0.02
2	554.01 ± 92.25	5748.11 ± 104.81	0.09 ± 0.01	1530.59 ± 195.74	7058.06 ± 102.53	0.18 ± 0.02
3	1729.37 ± 175.46	5536.84 ± 327.28	0.24 ± 0.01	3018.73 ± 90.28	6851.76 ± 109.34	0.31 ± 0.01
4	2846.72 ± 282.68	6534.97 ± 180.28	0.30 ± 0.02	3821.49 ± 329.36	6810.60 ± 461.51	0.36 ± 0.01
5	3542.26 ± 321.61	5489.51 ± 164.54	0.39 ± 0.01	6649.37 ± 294.44	7497.62 ± 376.96	0.47 ± 0.01
10	4788.00 ± 197.56	3822.85 ± 241.12	0.56 ± 0.01	11186.40 ± 363.43	5960.06 ± 476.54	0.65 ± 0.01
20	6433.83 ± 138.56	2672.44 ± 132.09	0.71 ± 0.01	11563.23 ± 336.65	3526.54 ± 268.74	0.77 ± 0.01
40	7190.57 ± 270.30	2199.01 ± 154.99	0.77 ± 0.01	10870.56 ± 253.99	2757.72 ± 179.35	0.80 ± 0.01
60	5879.72 ± 5.87	1657.17 ± 68.04	0.78 ± 0.01	9937.65 ± 248.34	1796.43 ± 250.67	0.85 ± 0.01
$[Ca^{2+}]$ (mM)	S-1A.1-a (Apo form)			S-1A.1-a (Holo form)		
	S1	F1	S1 Fraction	S1*	F1*	S1* Fraction
0	4.89 ± 2.93	6417.73 ± 216.31	0.01 ± 0.01	2.41 ± 0.00	11545.16 ± 529.20	0.01 ± 0.01
1	86.91 ± 1.97	8793.25 ± 57.92	0.01 ± 0.01	12.77 ± 1.29	6665.17 ± 288.03	0.01 ± 0.01
2	650.80 ± 141.44	6579.77 ± 73.59	0.09 ± 0.02	944.81 ± 34.47	5753.13 ± 263.08	0.14 ± 0.01
3	1541.70 ± 174.01	4959.49 ± 72.35	0.24 ± 0.02	3242.31 ± 271.13	4763.07 ± 277.98	0.40 ± 0.01
4	2682.91 ± 219.58	5156.21 ± 124.81	0.34 ± 0.01	3861.74 ± 118.00	10497.58 ± 244.44	0.27 ± 0.01
5	2512.21 ± 193.64	3848.30 ± 211.93	0.39 ± 0.01	6163.31 ± 113.02	4460.37 ± 130.30	0.58 ± 0.01
10	10666.09 ± 73.01	4575.53 ± 257.12	0.70 ± 0.01	6995.96 ± 210.30	3151.96 ± 768.51	0.69 ± 0.05
20	5928.60 ± 110.16	1778.31 ± 323.55	0.77 ± 0.03	7135.88 ± 241.38	1012.98 ± 147.54	0.88 ± 0.01
40	5829.07 ± 168.83	841.17 ± 219.93	0.87 ± 0.03	5150.92 ± 94.35	240.98 ± 21.62	0.96 ± 0.01
60	5017.85 ± 149.74	485.85 ± 88.63	0.91 ± 0.01	4665.07 ± 176.66	15.34 ± 16.06	1.00 ± 0.01

$[Mg^{2+}]$ (mM)	S-2.1-a (Apo form)			S-2.1-a (Holo form)		
	S2	F2	S2 Fraction	S2*	F2*	S2* Fraction
0	33488.09 ± 1325.24	38346.64 ± 3031.32	0.47 ± 0.02	21594.52 ± 1241.47	40678.90 ± 1486.26	0.35 ± 0.02
1	50659.13 ± 1280.55	19965.06 ± 1334.13	0.72 ± 0.01	46390.63 ± 542.50	19847.05 ± 558.04	0.70 ± 0.01
2	54242.13 ± 705.68	14109.47 ± 168.58	0.79 ± 0.01	44433.18 ± 2732.52	17525.16 ± 232.11	0.72 ± 0.02
3	53744.46 ± 828.45	12745.57 ± 748.06	0.81 ± 0.01	47098.38 ± 1144.22	19532.97 ± 3748.43	0.71 ± 0.04
4	53136.61 ± 1481.47	13112.65 ± 1418.43	0.80 ± 0.01	47743.07 ± 195.80	17374.20 ± 661.41	0.73 ± 0.01
5	51091.69 ± 522.84	18084.46 ± 1987.17	0.74 ± 0.02	46778.35 ± 742.20	16422.03 ± 405.19	0.74 ± 0.01
10	48218.68 ± 1656.77	24690.45 ± 2650.83	0.66 ± 0.03	44717.62 ± 1933.25	14968.19 ± 553.62	0.75 ± 0.01
20	45072.32 ± 582.43	21682.26 ± 1265.35	0.68 ± 0.01	42083.97 ± 464.96	13749.41 ± 309.28	0.75 ± 0.01
40	42714.91 ± 487.80	10001.81 ± 1310.83	0.81 ± 0.02	33659.13 ± 160.48	10194.96 ± 370.30	0.77 ± 0.01
60	47998.03 ± 8926.25	4467.53 ± 1601.19	0.91 ± 0.04	39965.78 ± 35.15	11337.63 ± 83.76	0.78 ± 0.01

$[Ca^{2+}]$ (mM)	S-2.1-a (Apo form)			S-2.1-a (Holo form)		
	S2	F2	S2 Fraction	S2*	F2*	S2* Fraction
0	13579.52 ± 342.66	33849.51 ± 1740.89	0.29 ± 0.01	6558.90 ± 427.74	8161.51 ± 138.27	0.45 ± 0.02
1	30965.33 ± 522.45	10785.70 ± 40.13	0.74 ± 0.01	14949.78 ± 770.72	4861.42 ± 583.23	0.76 ± 0.01
2	32970.98 ± 233.17	7830.78 ± 95.98	0.81 ± 0.01	16923.54 ± 491.20	3358.14 ± 542.27	0.84 ± 0.02
3	29070.27 ± 276.96	5568.86 ± 170.21	0.84 ± 0.01	18238.19 ± 230.74	3004.69 ± 440.70	0.86 ± 0.02
4	29386.87 ± 404.72	7083.75 ± 153.29	0.81 ± 0.01	12322.02 ± 148.51	4997.43 ± 117.43	0.71 ± 0.01
5	29264.90 ± 61.52	5824.83 ± 368.38	0.83 ± 0.01	19347.30 ± 96.41	2125.46 ± 322.70	0.90 ± 0.01
10	35555.81 ± 416.13	6402.67 ± 397.81	0.85 ± 0.01	19827.50 ± 145.17	1928.24 ± 217.67	0.91 ± 0.01
20	30614.31 ± 748.63	5074.71 ± 365.19	0.86 ± 0.01	20732.92 ± 194.92	1014.43 ± 154.34	0.95 ± 0.01
40	25579.55 ± 222.99	5437.12 ± 106.81	0.82 ± 0.01	20173.69 ± 229.41	713.10 ± 111.90	0.97 ± 0.01
60	23294.02 ± 355.88	3927.23 ± 151.28	0.86 ± 0.01	18524.17 ± 190.37	391.63 ± 74.93	0.98 ± 0.01
